# Impaired nick recognition and ligation efficiency by LIG1 K845N variant linked to Huntington’s disease

**DOI:** 10.1093/narmme/ugaf038

**Published:** 2025-10-28

**Authors:** Jacob Ratcliffe, Camden M Lerner, Kanal Balu, Surajit Chatterjee, Kar Men Lee, Melike Caglayan

**Affiliations:** Department of Biochemistry and Molecular Biology, University of Florida, Gainesville, FL 32610, United States; Department of Biochemistry and Molecular Biology, University of Florida, Gainesville, FL 32610, United States; Department of Biochemistry and Molecular Biology, University of Florida, Gainesville, FL 32610, United States; Department of Biochemistry and Molecular Biology, University of Florida, Gainesville, FL 32610, United States; Department of Biochemistry and Molecular Biology, University of Florida, Gainesville, FL 32610, United States; Department of Biochemistry and Molecular Biology, University of Florida, Gainesville, FL 32610, United States

## Abstract

DNA ligase 1 (LIG1) joins broken strand breaks and seals Okazaki fragments during DNA repair and replication. Huntington’s disease (HD)-associated mutation in the *LIG1* gene, K845N, is associated with delayed symptom onset and predicted to suppress CAG repeat expansion. Yet, how this mutation impacts faithful nick sealing and efficient DNA binding by LIG1 remains unknown. Here, using biochemical analyses, X-ray crystallography, and total internal reflection fluorescence (TIRF) microscopy, we characterized the LIG1 HD-associated K845N variant at biochemical, structural, and single-molecule levels. Our results showed significantly reduced ligation efficiency for nick substrates containing noncanonical mismatches and diminished mutagenic end-joining of damaged DNA, while LIG1 K845N variant exhibits a lack of discrimination against nicks containing 3′-ribonucleotides when compared with the wild-type enzyme. Furthermore, our structures provided an atomic insight into differences in the distances between functional groups of K/N845 and DNA ends, demonstrating similar conformation at the ligase active site. Finally, our single-molecule measurements revealed that the K845N variant binds less frequently to nick, suggesting diminished affinity. Overall, our findings contribute to understanding the mechanism by which LIG1 searches for nick sites on DNA and ensures fidelity to maintain genome stability at the final ligation step in normal versus HD-associated states.

## Graphical Abstract

**Graphical Abstract:**
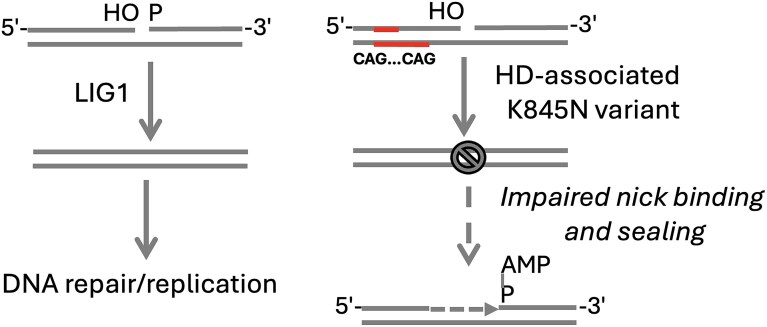

## Introduction

Mammalian genomes are exposed to multiple types of DNA damage that can be generated during endogenous cellular processes and by exogenous sources such as environmental factors including UV light, chemical toxins, irradiation, inflammation, and nutritional factors [1]. The combined actions of DNA replication machinery and a network of DNA repair pathways are essential for maintaining genome stability [2]. DNA ligases seal nicks to repair broken strand breaks during these criticial DNA transactions [3]. ATP-dependent human DNA ligases catalyze a phosphodiester bond formation between the 3′-hydroxyl (OH) and 5′-phosphate (PO_4_) ends of a nick in a conserved three-step of DNA ligation reaction [4, 5]. In the first step, the DNA ligase reacts with a nucleotide cofactor (ATP) and releases a pyrophosphate group, forming a covalent ligase-adenylate intermediate. In the second step, an adenosine monophosphate (AMP) moiety is transferred from the adenylated ligase to the 5′-PO_4_ end of nick. In the final step of the ligation reaction, the 3′-OH acts as a nucleophile, attacking the 5′-PO_4_, which releases AMP.

Of the three human DNA ligases, DNA ligase 1 (LIG1) is the main replicative ligase playing a critical role in the maturation of Okazaki fragments during DNA replication and finalizes DNA excision repair pathways with an ultimate nick sealing step [6–8]. The expression level of LIG1 was found higher in human cancer cells, and the ligase is essential for embryonic development, but some studies have demonstrated that LIG1 is not required for cellular DNA replication and that either LIG3α or LIG4 can substitute for joining Okazaki fragments [9–11].

The fidelity of ligation at the final step of DNA repair pathways requires a Watson–Crick base-paired end at the nick (i.e. 3′-dG:C) after a correct nucleotide incorporation into a gap repair intermediate by DNA polymerase (i.e. dGTP:C) during the DNA synthesis step [12, 13]. In our previous studies, we demonstrated the aberrant repair due to impared LIG1 and LIG3α activities at the downstream steps of base excision repair (BER) pathway [14–21]. Particularly, after incorporation of mismatches or oxidized nucleotides (i.e. 8-oxodGTP) by DNA polymerase (pol) β, the resulting nick product cannot be efficiently sealed by both BER ligases leading to ligase failure and formation of deleterious repair intermediates with a 5′-adenylate (AMP) block [14–18]. These abortive ligation products can be then cleaned by the DNA-end processing enzymes such as Aprataxin (APTX) and Flap Endonuclease 1 (FEN1) [19]. Furthermore, our LIG1 structures demonstrated the strategies that the ligase active site utilizes to discriminate against nicks containing mismatches depending on the architecture of 3′-terminus:template base-pairing, and oxidatively damaged ends (8oxoG) depending on the dual coding potential of the oxidative lesion in -*anti* versus -*syn* conformation [22, 23]. Furthermore, in our structures of LIG1/RNA–DNA hybrids, we demonstrated that the ligase shows a lack of discrimination against nicks containing a single ribonucleotide at the 3′-end, and can seal almost all possible 3′-ribonucleotide-containing mismatches as efficient as nicks with Watson–Crick base-paired ends [24, 25]. These studies contributed to understand the mechanism by which LIG1 ensures fidelity while engaging with potentially mutagenic and toxic repair intermediates during nuclear replication and at the final ligation step of DNA repair pathways. However, it’s still largely unknown how pertubrations at the ligase active site, particularly disease-associated mutations in the catalytic core of LIG1, could impact the efficiency of initial nick DNA binding, accuracy of nick sealing, and ligase fidelity.

Polyglutamine disorders are inheritable and are caused by the expansion of cytosine–adenine–guanine (CAG) repeats in DNA [26]. Huntington’s disease (HD) is the most prominent of the polyglutamine diseases as a dominantly inherited neurological disorder, characterized by an array of symptoms such as cognitive decline, involuntary movements, and changes to behavior and speech [27]. The expansion of CAG repeats in the Huntingtin (HTT) gene leads to generation of the toxic Huntingtin protein (mHTT), and its build up disrupts neuronal function causing neurodegeneration [28]. HD is caused by unstable repeat expansion and lengthening of the trinucleotide repeat CAG [29]. The Genetic Modifiers of HD (GeM-HD) Consortium utilized a genome wide association study (GWAS) of over 9000 individuals with HD and reported that age of HD onset is determined by CAG repeat length regardless of polyglutamine length, demonstrating a relationship between CAG repeats and HD symptom onset [30]. It has been reported in this study that the chromosome 19, which encodes LIG1, harbors a rare modifier haplotype (19AM3) that is strongly onset delaying and is tagged by the LIG1 missense change K845N [30]. Yet, it remains unknown how the HD-associated K845N mutation in the *LIG1* gene could impact the efficiency of nick binding and whether the K845N variant can affect the mechanism of discrimination against noncanonical nicks that mimic base-substitution errors or potentially mutagenic repair intermediates introduced by repair and replication DNA polymerases. Importantly, LIG1 is involved in CAG repeat expansion following polβ-mediated repeat synthesis during BER of the oxidative damage due to the formation of a DNA hairpin structure that prevents FEN1 from cleaving the repeat sequence leading to aberrant repair [31]. Thus, understanding how DNA ligation is impacted by the K845N mutation is critical to elucidate how LIG1 ensures fidelity at the final step of DNA repair in normal versus disease states, which could contribute to explain the mechanism behind the observed symptom onset delay and suppression of repeat expansion in HD.

In the present study, using a combined approach including biochemistry, X-ray crystallography, and single-molecule fluorescence microscopy, we explored the impact of the LIG1 HD-associated mutation K845N on the efficiency of nick binding and ligation at the biochemical, structural, and single-molecule levels. For this purpose, we compared DNA end-joining ability of LIG1 wild-type and K845N variant for a range of nick substrates containing canonical, mismatched, damaged, and ribonucleotide-containing ends *in vitro*. Our findings demonstrated that the ligation efficiency is reduced by the effect of K845N mutation compared to wild-type enzyme, and the LIG1 variant exhibits an increased discrimination against nicks containing mismatches and oxidatively damaged ends. Furthermore, the X-ray structure of LIG1 in the absence and presence of the K845N mutation showed a similar DNA conformation and distances relative to the 3′- and 5′-ends of nick. Finally, our single-molecule DNA-binding measurements in real-time demonstrated that the K845N mutant protein binds less frequently to nick compared to LIG1 wild-type. Overall, our findings provide mechanistic insights into the mechanism by which the accuracy of nick sealing and ligase fidelity could be impaired by the LIG1 HD-associated K845N mutation during DNA repair and replication. This study could contribute to elucidate the biochemical mechanism of the neurodegenerative symptoms caused by HD and biochemical defects observed in LIG1 pathological variant underscore LIG1 function in distinct disease states.

## Materials and methods

### Protein purification

We generated the Lys(K) to Asn(N) amino acid substitution at the residue 845 of LIG1, which resides in the oligonucleotide-binding domain (OBD) of the catalytic core (Supplementary Fig. S1A). We generated the K845N mutation in the C-terminal (△261) and full-length (1–919 amino acids) background (pET-24b) of LIG1. The recombinant proteins, LIG1 wild-type and K845N variant, with 6x his-tag were purified as described previously [14–25]. Briefly, the proteins were overexpressed in *Escherichia coli* (DE3) cells and grown in Terrific Broth (TB) media with kanamycin (50 μg ml^−1^) and chloramphenicol (34 μg ml^−1^) at 37°C. Once the OD_600_ reached 1.0, cells were induced with 0.5 mM isopropyl β-d-thiogalactoside (IPTG) and overexpression continued overnight at 20°C. After centrifugation, the cells were lysed in the lysis buffer containing 50 mM Tris–HCl (pH 7.0), 500 mM NaCl, 20 mM imidazole, 2 mM β-mercaptoethanol, 5% glycerol, and 1 mM phenylmethylsulfonyl fluoride (PMSF) by sonication at 4°C. The cell lysate was pelleted at 31 000 × *g* for 90 min at 4°C. The cell lysis solution was clarified and then loaded onto a HisTrap HP column that was previously equilibrated with the binding buffer containing 50 mM Tris–HCl (pH 7.0), 500 mM NaCl, 20 mM imidazole, 2 mM β-mercaptoethanol, and 5% glycerol. The column was washed with the binding buffer and then eluted with an increasing imidazole gradient 20–500 mM at 4°C. The collected fractions were then subsequently loaded onto a HiTrap Heparin column that was equilibrated with the binding buffer containing 20 mM Tris–HCl (pH 7.0), 50 mM NaCl, 2 mM β-mercaptoethanol, and 5% glycerol. The protein was eluted with a linear gradient of NaCl up to 1 M. LIG1 proteins were further purified by a Superdex 200 10/300 column in the buffer containing 50 mM Tris–HCl (pH 7.0), 200 mM NaCl, and 1 mM dithiothreitol (DTT), and 5% glycerol. LIG1 proteins purified in this study were concentrated, aliquoted, and stored at −80°C. Protein quality was evaluated on a 10% sodium dodecyl sulfate–polyacrylamide gel electrophoresis (SDS–PAGE) gel, and protein concentrations were measured using absorbance at 280 nm (Supplementary Fig. S1B).

### DNA ligation assays

Nick DNA substrates with a 6-carboxyfluorescein (FAM) label were used in DNA ligation assays. Nick DNA substrates containing Watson–Crick base-paired ends, and all 12 noncanonical mismatches were prepared by annealing upstream oligonucleotides 3′-dA, dT, dG, or dC and downstream oligo containing 3′-FAM with template oligonucleotides A, T, G, or C (Supplementary Table S1). Nick DNA substrates containing damaged ends were prepared by annealing upstream oligonucleotide 3′-8oxodG and downstream oligo containing 3′-FAM with template oligonucleotides A or C (Supplementary Table S2). Nick DNA substrates containing a single ribonucleotide at the 3′-end were prepared by annealing upstream oligonucleotides 3′-rA, 3′-rG, or 3′-rC and downstream oligo containing 3′-FAM with template oligonucleotides A, T, G, or C (Supplementary Table S3). Nick DNA substrates containing CAG repeats were prepared by annealing upstream oligonucleotide 3′-dG and downstream oligonucleotides 3′-CAG or 5′-CAG containing 3′-FAM with template oligonucleotide C (Supplementary Table S4). DNA ligation assays were performed as described [14–25] to investigate the substrate specificity of LIG1 wild-type and K845N mutant for the nick DNA substrates containing canonical, mismatched, damaged, and ribonucleotide-containing ends as well as CAG repeats (Supplementary Schemes S1 and 2). The reaction was initiated by the addition of LIG1 (100 nM) to a mixture containing 50 mM Tris–HCl (pH 7.5), 100 mM KCl, 10 mM MgCl_2_, 1 mM ATP, 1 mM DTT, 100 µg ml^−1^ bovine serum albumin (BSA), 1% glycerol, and the nick DNA substrate (500 nM) in a final volume of 10 µl. The reaction mixture was incubated at 37°C and stopped at the time points indicated in the figure legends by mixing with an equal volume of loading dye containing 95% formamide, 20 mM ethylenediaminetetraacetic acid, 0.02% bromophenol blue, and 0.02% xylene cyanol. Reaction products were separated by electrophoresis on an 18% Urea–PAGE gel, the gels were scanned with the Typhoon PhosphorImager RGB, and the data were analyzed using ImageQuant software. Graphs showing ligation product over time were drawn, and the data were analyzed by two-way ANOVA with multiple comparisons using GraphPad Prism 10. When *P*-values were < 0.05, data were deemed statistically significant.

### Crystallization and structure determination

For the crystallization of LIG1 K845N, we used LIG1 C-terminal (△261) harboring the EE/AA (E346A and E592A) mutations, resulting in the ablation of the high-fidelity site, which has been utilized in our previous LIG1 structures with noncanonical nicks [22–25]. We solved the structure of LIG1 K845N in complex with nick DNA containing a canonical (A:T) end (Supplementary Table S5). LIG1 (at 27 mg ml^−1^)/DNA complex solution was prepared in the buffer containing 20 mM Tris–HCl (pH 7.0), 200 mM NaCl, 1 mM DTT, 1 mM EDTA, and 1 mM ATP at 1.4:1 DNA:protein molar ratio and then mixed with 1 μl reservoir solution. LIG1–nick DNA complex crystals were grown at 20°C using the hanging drop method, harvested, and submerged in the cryoprotectant solution containing reservoir solution mixed with glycerol to a final concentration of 20% glycerol before being flash cooled in liquid nitrogen (Supplementary Table S6). LIG1 crystals were obtained and kept at 100 K during X-ray diffraction data collection using the beamline CHESS-7B2. The collected data were reduced and scaled using HKL2000 (HKL Research, Inc). LIG1 K845N structure was solved by the molecular replacement method using PHASER with PDB entry 7SUM as a search model [32]. Iterative rounds of model building were performed in COOT and the final models were refined with PHENIX [33, 34]. All structural images were drawn using PyMOL (The PyMOL Molecular Graphics System, V0.99, Schrödinger, LLC). Detailed crystallographic statistics are provided in Table 1.

**Table 1.** X-ray data collection and refinement statistics for the structure of LIG1 K845N variant

	LIG1 K845N
**Data collection**	
Space group	P2_1_2_1_2_1_
Cell dimensions	
*a, b, c* (Å)	74.7 100.1, 115.1
α, β, γ (°)	90
Resolution (Å)	300–2.64 (2.80–2.64)
*R*_meas_	0.38 (1.05)
*I*/σ (*I*)	14.2 (4.0)
CC_1/2_	0.995 (0.887)
Completeness (%)	99.7 (99.0)
Redundancy	13.2 (13.1)
**Refinement**	
Resolution (Å)	30–2.64
No. reflections	25 820
*R*_work_/*R*_free_	0.178/0.2236
Non-H atoms:	5867
Protein	4876
DNA	733
AMP	22
Solvent	216
Average *B*-factors (Å^2^):	35.86
Protein	36.80
DNA	32.79
AMP	45.46
Solvent	34.58
R.M.S.D	
Bond lengths (Å)	0.003
Bond angles (°)	0.565

Values in the paranthesis are for the highest resolution shell.

### Single-molecule nick DNA-binding measurements

We performed single-molecule imaging experiments to monitor LIG1–nick DNA binding using a total internal reflection fluorescence (TIRF) microscopy (Nikon Eclipse Ti2-E) as reported previously [20]. Briefly, we fluorescently labeled LIG1 full-length proteins (wild-type and K845N variant) with Amersham Cy5 monoreactive NHS ester dye (LIG1^Cy5^) and used 3′-AF488-labeled dsDNA substrate (34-mer) including 5′-biotin and a single nick site containing a canonical A:T or damaged 8oxoG:A as well as 3′-AF488-labeled dsDNA substrate including 5′-biotin and no nick site (Supplementary Table S7). Glass coverslips were functionalized with a mixture of biotin-PEG-SVA and mPEG-SVA and the microfluidic channels were assembled using the passivated coverslips and Grace Bio-Labs HybriWell^™^ sealing system, which were rinsed three times with the T50 buffer containing 10 mM Tris–HCl (pH 8.0) and 50 mM NaCl. Streptavidin (0.2 mg/ml) in the T50 buffer was then flowed onto the slide, reacted with the biotin-PEG, and then washed with the T50 buffer. DNA substrate was diluted to a final concentration of 10 pM in the imaging buffer containing 1 mM HEPES (pH 7.4), 20 mM NaCl, 0.02% BSA (w/v), and 0.002% Tween 20 (v/v), flowed onto the slide, and an excess unbound DNA substrate was then washed by flowing with the imaging buffer. Oxygen-scavenging system (OSS) consisting of 44 mM glucose, 165 U/ml glucose oxidase from Aspergillus niger and catalase (2 170 U/ml) were added to slow photobleaching. 10 mM Trolox was added to reduce photo blinking. Finally, LIG1^Cy5^ proteins (1 nM) in the imaging buffer containing the OSS mixture were flowed onto the slide and allowed to equilibrate before imaging with an objective-based TIRF microscope. Both AF488 and Cy5 dyes were simultaneously excited using 488 and 640 nm lasers (power 5 mW at the source), respectively. All imaging experiments were performed at room temperature. Emissions from two fluorophores were separated into two channels using a Cairn Optosplit II image splitter and simultaneously recorded at 100 ms time resolution using a Hamamatsu SCMOS camera (77054115) using NIS-Elements acquisition software (Nikon, version: AR 6.02.01). The locations of molecules and fluorophore intensity over time traces were extracted from the raw movie files using Nikon NIS-Elements analysis software (Nikon, version: AR 6.02.01). Genuine fluorescence time traces for individual molecules were selected using NIS-Elements time measurement analysis and were idealized using a two-state hidden Markov model (HMM) for the unbound and bound states in QuB. Rastergrams summarizing several individual traces were generated from the individual trace HMMs using a custom written MATLAB script. From the idealized traces, dwell times of the bound and unbound states were calculated using MATLAB. Cumulative frequency of the bound and unbound dwell-time distributions was plotted and fitted in Origin Lab (version 2024b) with single or double exponential functions to obtain the bound (*t*_bound_) and unbound (*t*_unbound_) state lifetimes.

## Results

### Impact of the LIG1 K845N mutation on the ligase fidelity against nicks containing a ribonucleotide, mismatched or damaged base at the 3′-end

We comprehensively characterized the ligation profiles of LIG1 wild-type and K845N variant for all 12 possible noncanonical nicks containing mismatches or a single ribonucleotide as well as nicks harboring a damaged base (8-oxoG) at the 3′-end. These substrates are biologically relevant, and mimic potentially mutagenic repair intermediates that can be generated due to DNA polymerase-mediated errors during DNA repair and replication [12, 13].

**Figure 1.**
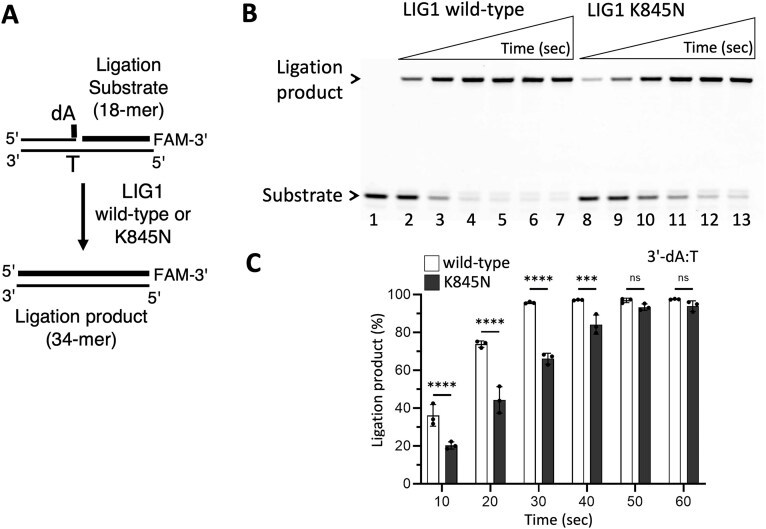
Ligation efficiency of canonical nick DNA by LIG1 K845N variant. (**A**) Scheme shows ligase substrate and reaction product observed in the ligation assay in the presence of nick substrate containing a canonical 3′-dA:T. (**B**) Line 1 is the negative enzyme control of the ligation reaction including nick DNA substrate alone. Lanes 2–7 and 8–13 are the ligation reaction products by LIG1 wild-type and K845N variant, respectively, and correspond to time points of 10, 20, 30, 40, 50, and 60 s. (**C**) Graph shows time-dependent changes in the amount of ligation products. Data points represent three independent replicates. Bar height is the mean, and error bars represent the SD. n.s., not significant; ^****^P < 0.0001 by ordinary two-way ANOVA with multiple comparisons.

**Figure 2.**
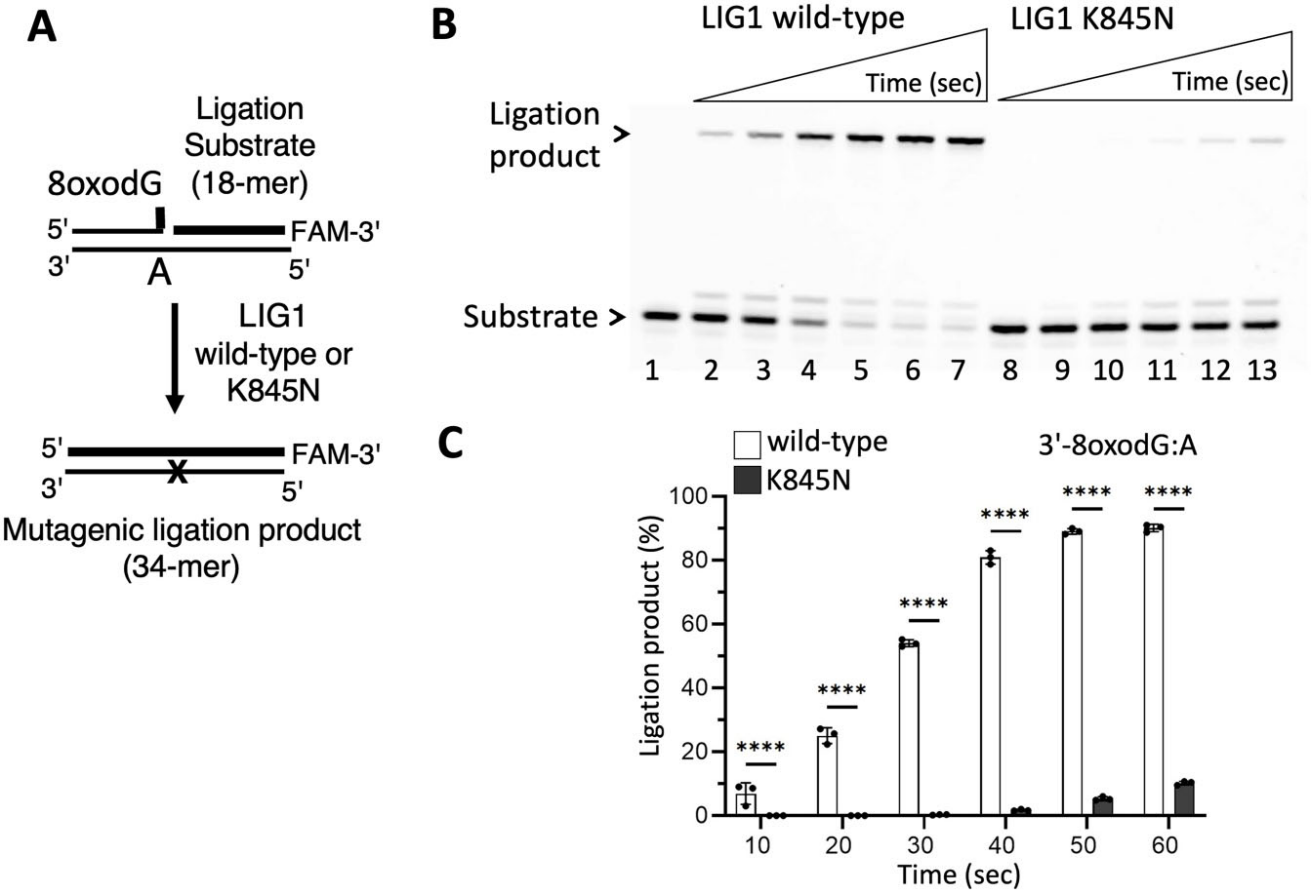
Impact of LIG1 K845N HD-associated mutation on the ligase fidelity. (**A**) Scheme shows ligase substrate and reaction product observed in the ligation assay in the presence of nick substrate containing 3′-8oxoG:A. (**B**) Line 1 is the negative enzyme control of ligation reaction including nick DNA substrate alone. Lanes 2–7 and 8–13 are the ligation reaction products by LIG1 wild-type and K845N variant, respectively, and correspond to time points of 10, 20, 30, 40, 50, and 60 s. (**C**) Graph shows time-dependent changes in the amount of ligation products. Data points represent three independent replicates. Bar height is the mean, and error bars represent the SD. n.s., not significant; ^****^P < 0.0001 by ordinary two-way ANOVA with multiple comparisons.

In the presence of nick substrate containing a canonical end (3′-dA:T), our results demonstrated less efficient ligation and reduced amount of nick sealing products by the LIG1 K845N variant when compared to the wild-type enzyme (Fig. 1). Furthermore, we showed significantly compromised mutagenic ligation of the nick DNA substrate containing 3′-8oxodG:A by LIG1 K845N variant, suggesting a role of the K845 residue for discriminating against nicks with damaged ends and its contribution to maintan fidelity at the last ligation step of DNA repair pathways (Fig. 2).

**Figure 3.**
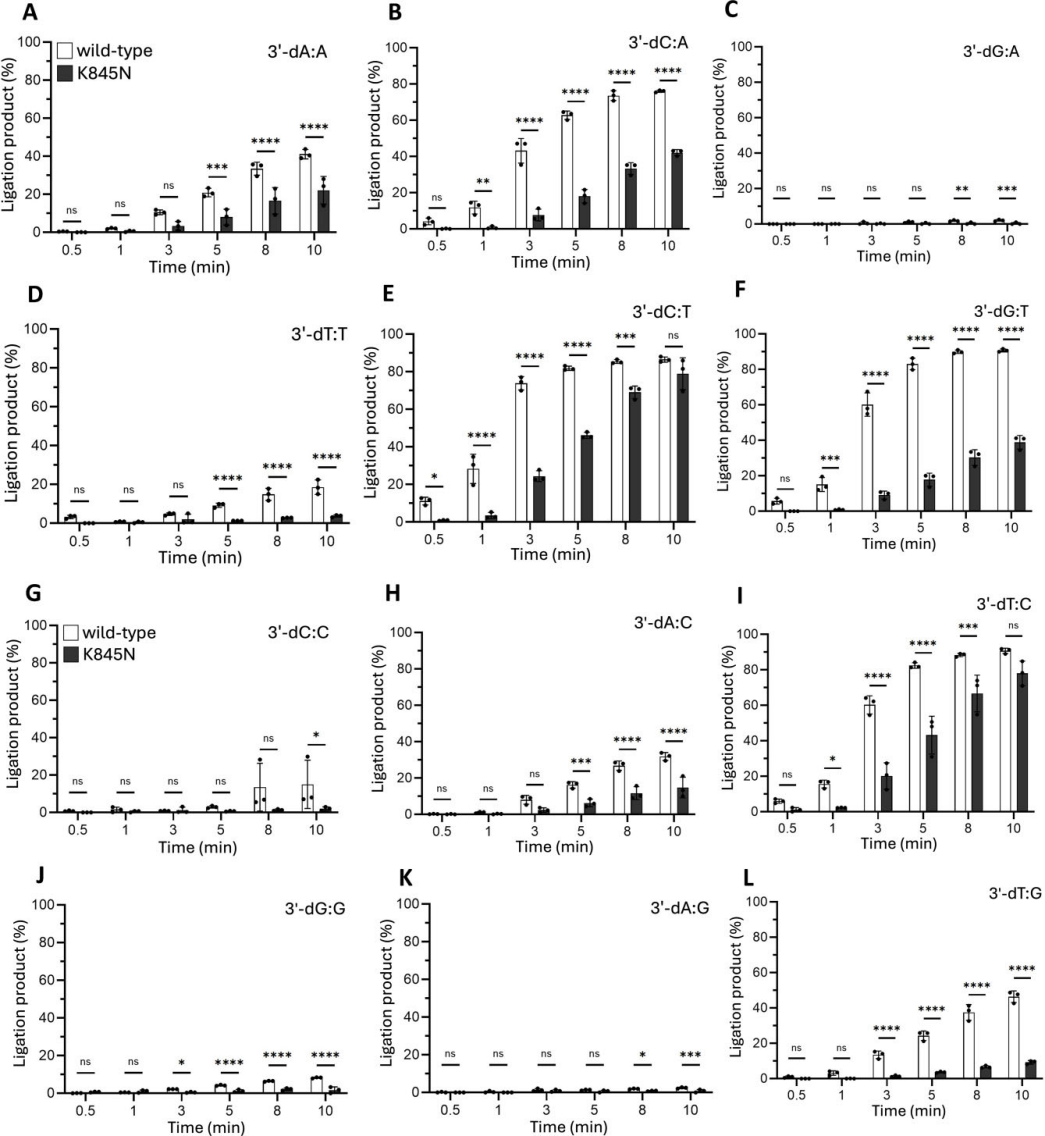
Ligation efficiency of LIG1 wild-type versus K845N variant for nick DNA substrates containing all 12 noncanonical 3′-mismatches. (**A–L**) Graphs show time-dependent changes in the amount of ligation products. Data points represent three independent replicates. Bar height is the mean, and error bars represent the SD. n.s., not significant; *P < 0.05, **P < 0.01, ***P < 0.001, ^****^P < 0.0001 by ordinary two-way ANOVA with multiple comparisons. Representative gel images showing time-dependent ligation product formation for all 3′-mismatches are presented in Supplementary Figs S2 and 3.

We then compared end joining abilities of LIG1 wild-type and K845N variant for a range of nick substrates containing all 12 possible 3′-mismatches. Our results showed that both LIG1 proteins exhibit subtle differences in the ligation efficiency depending on the architecture of the 3′-terminus:template base pairing (Fig. 3, and Supplementary Figs S2 and 3). Particularly, in the presence of 3′-dC:A, 3′-dC:T, 3′-dG:T, 3′-dT:C, and 3′-dT:G mismatches, there was relatively less efficient nick sealing, while both LIG1 proteins showed a similar ligation profile for nicks with 3′-dA:A and 3′-dA:C mismatches. However, the nick DNA containing the purine:purine mismatches, 3′-dG:A and 3′-dA:G, cannot be ligated by either LIG1 protein. Similarly, we observed relatively less efficient ligation in the presence of nick DNA substrates containing 3′-dC:C and 3′-dT:T mismatches. For the same time points of reaction incubation with all 12 noncanonical mismatches, we observed a significant decrease in the ligation efficiency for the nick substrates containing 3′-8oxodG:A and 3′-8oxodG:C by the LIG1 K845N variant, although LIG1 wild-type was able to efficiently seal these nick repair intermediates (Supplementary Fig. S4A–D). Our results demonstrated no ligation product in the presence of nick substrate containing a nonmutagenic 3′-8oxodG:C base-pairing by the effect of K845N mutation, while LIG1 wild-type exhibits a preference for mutagenic ligation of 8oxoG paired with A over C (Supplementary Fig. S4E and F). Overall comparison of ligation profiles between LIG1 K845N and wild-type demonstrates significantly less efficient ligation as well as discrimination against sealing nicks containing particular mismatches and damaged ends.

**Figure 4.**
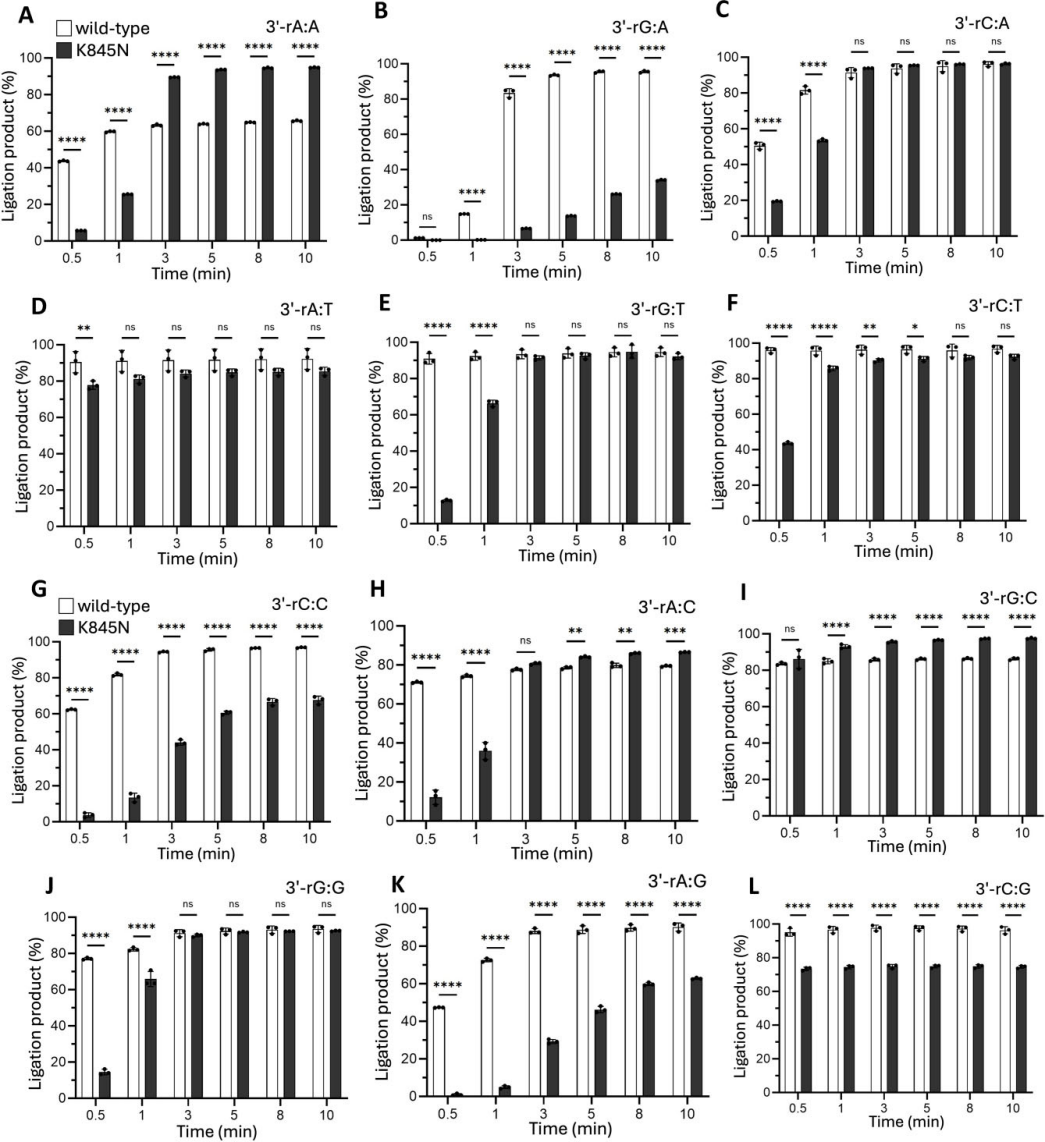
Ligation efficiency of LIG1 wild-type versus K845N variant for nick DNA substrates containing a single ribonucleotide 3′-end. (**A**–**L**) Graphs show time-dependent changes in the amount of ligation products. Data points represent three independent replicates. Bar height is the mean, and error bars represent the SD. n.s., not significant; *P < 0.05, **P < 0.01, ***P < 0.001, ^****^P < 0.0001 by ordinary two-way ANOVA with multiple comparisons. Representative gel images showing time-dependent product formation are presented in Supplementary Figs S5 and 6.

Furthermore, we investigated the ligation profile of LIG1 wild-type and K845N variant in the presence of a “wrong sugar” at the 3′-end of nick DNA substrate to further understand the impact of the HD-associated mutation on the mechanism of sugar discrimination by LIG1. For this purpose, we used the nick DNA substrates containing all possible 12 mismatches containing a single ribonucleotide at the 3′-end that mimic the ribonucleotide insertion products of DNA polymerases before LIG1 seals the resulting nick intermediate during Okazaki fragment maturation of DNA replication or at the last ligation step of DNA repair pathways [12, 13]. Our results with LIG1 wild-type and K845N variant demonstrated a lack of sugar discrimination as both proteins were able to ligate nick DNA substrates with 3′-preinserted ribonucleotide mismatches, especially for the Watson–Crick base-paired nick DNA substrates containing 3′-rA:T, 3′-rC:G, and 3′-rG:C (Fig. 4 and Supplementary Figs S4–6). However, in the presence of nick substrates containing 3′-rA:G, 3′-rG:A, and 3′-rA:G mismatches, there was significantly less efficient ligation by the LIG1 K845N variant in comparison with LIG1 wild-type. In general, we observed a robust accumulation of ligation products over reaction time, suggesting no impact of the K845N mutation on the sugar discrimination mechanism of LIG1.

**Figure 5.**
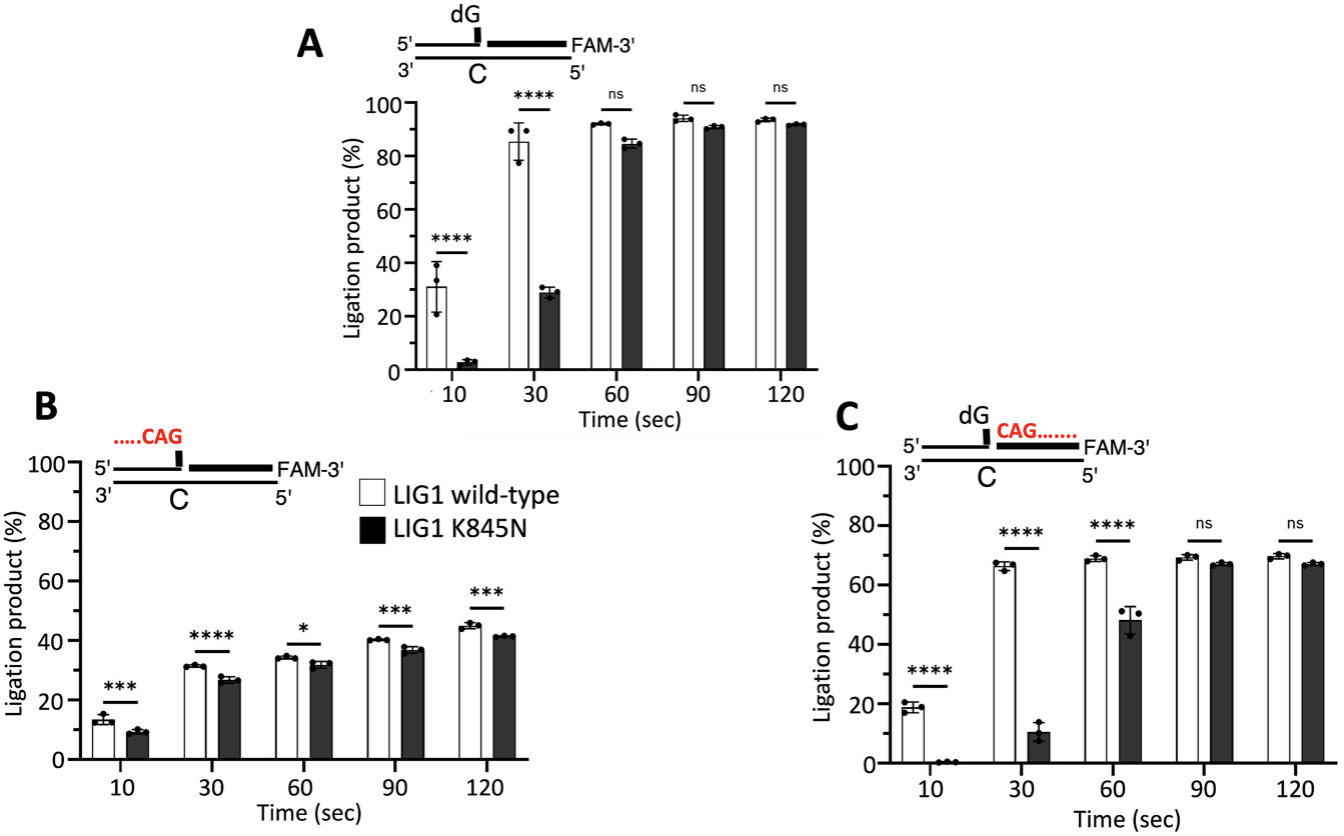
Ligation efficiency of LIG1 K845N wild-type versus K845N variant for nick DNA substrates containing CAG repeats. (**A**–**C**) Graphs show time-dependent changes in the amount of ligation products. Data points represent three independent replicates. Bar height is the mean, and error bars represent the SD. n.s., not significant; *P < 0.05, ***P < 0.001, ^****^P < 0.0001 by ordinary two-way ANOVA with multiple comparisons. Representative gel images showing time-dependent product formation are presented in Supplementary Fig. S7.

Lastly, we explored the impact of the K845N mutation on ligation efficiency in the presence of the nick DNA substrates relevant to repeat expansion disorders, specifically HD. We, therefore, compared the end-joining ability of LIG1 wild-type and K845N variant for the nick DNA substrates containing CAG repeats at the upstream or downstream position relative to the nick site (Fig. 5). Our results demonstrated that the LIG1 K845N mutation significantly decreases ligation efficiency on both substrates, with the effect being more dramatic on the downstream CAG substrate (Supplementary Fig. S7). However, interestingly, both LIG1 proteins were less efficient at ligating these substrates compared to the canonical control. This highlights both the effect of CAG repeat presence and positioning on ligation efficiency by both LIG1 proteins.

### Structure of LIG1 K845N in complex with a canonical nick

In addition to a comprehensive biochemical characterization of the LIG1 K845N mutant in the ligation assays, we determined its structure in complex with nick DNA containing a canonical A:T (Table 1). For this purpose, we used the LIG1 low-fidelity EE/AA mutant that harbors E346A and E592A mutations (LIG1^EE/AA^) for crystallization of the triple mutant LIG1^EE/AA^K845N. We used this low-fidelity mutant in our previously reported LIG1 structures [22–25]. In the ligation asssays, we demonstrated no significant difference in the ligation efficiency between LIG1^EE/AA^ double-mutant and LIG1^EE/AA^ K845N triple-mutant (Supplementary Fig. S8). We used our previously solved structure of LIG1^EE/AA^/A:T for comparison with LIG1^EE/AA^K845N [22].

**Figure 6.**
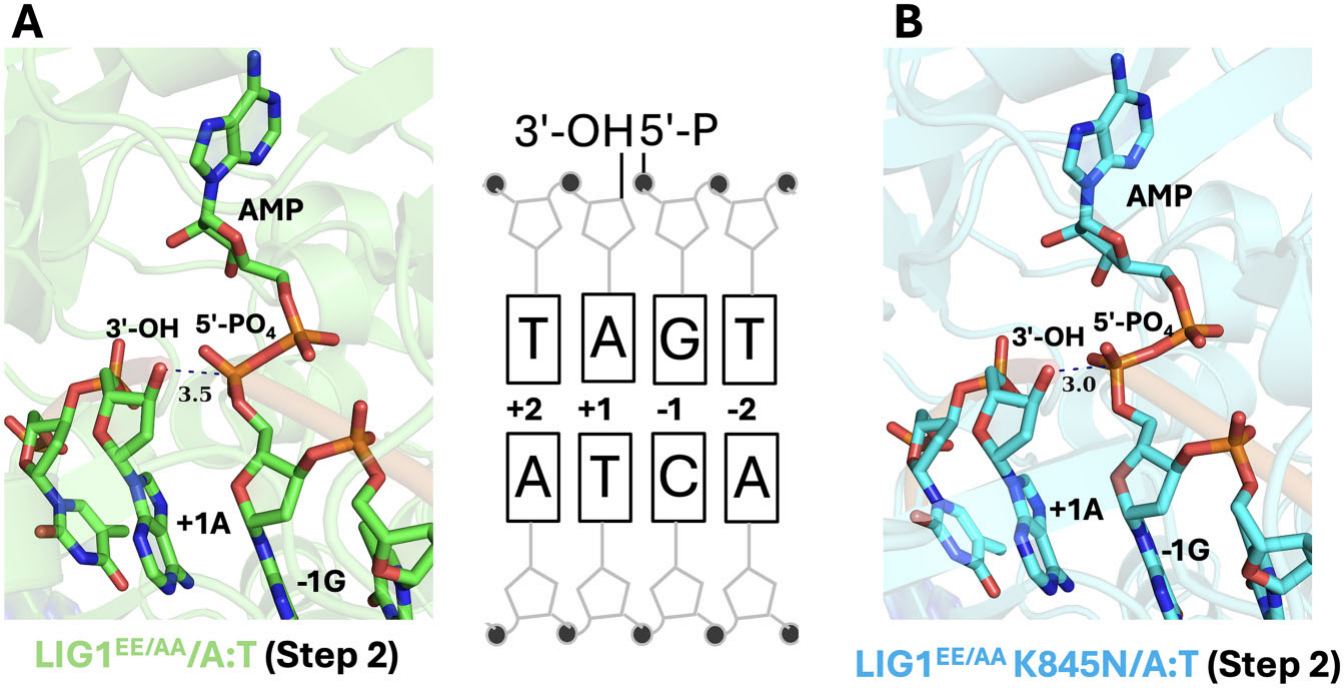
Structure of LIG1 K845N bound to nick DNA. (**A** and **B**) Structures of LIG1^EE/AA^ and LIG1^EE/AA^K845N show step 2 of the ligation reaction where AMP is bound to 5′-PO_4_ end of nick. The 2Fo-Fc density map of the AMP is contoured at 1.5σ (blue). LIG1 is shown in cartoon mode and DNA, AMP, active site residues are shown in stick mode. Scheme shows the sequence of nick DNA substrate used in LIG1 crystallization. LIG1^EE/AA^/3′-dA:T structure is previously solved (PDB: 7SUM).

**Figure 7.**
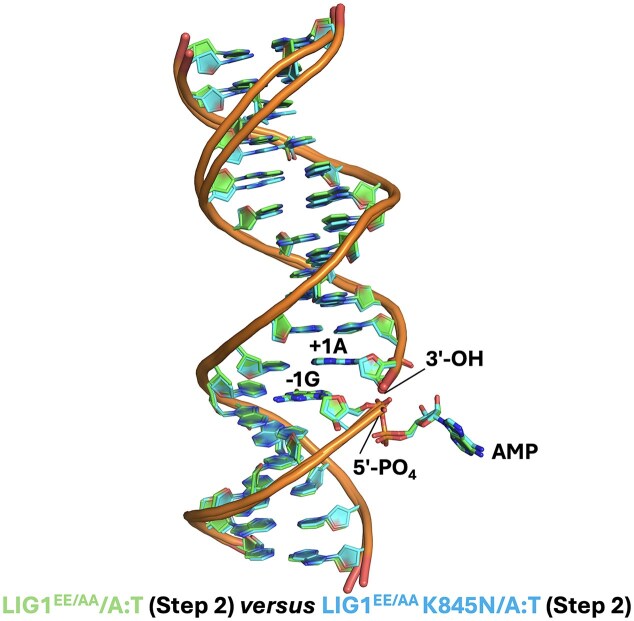
Superimposition of LIG1 structures with and without K845N mutation. Overlay of LIG1^EE/AA^ and LIG1^EE/AA^K845N structures in complex with nick DNA containing canonical A:T end shows a similarity in the DNA conformation at the downstream of the nick.

In the structure of LIG1^EE/AA^/A:T, we observed that an AMP moiety is bound to the 5′-end of the nick where the DNA–AMP intermediate is formed, which refers to step 2 of the ligation reaction (Fig. 6A). Similarly, in the structure of LIG1^EE/AA^ K845N/A:T, we observed the ligase active site at step 2 of the ligation reaction (Fig. 6B). The overlay of both LIG1 structures demonstrated similar confirmation with a root mean square deviation (RMSD) of 0.560Å. The superimposition of these structures also shows a similarity in the DNA conformation at the downstream of the nick in the absence and presence of the K845N mutation residing in the OBD domain (Fig. 7). We did not observe any large scale alternations at the ligase active site due to the K845N mutation.

**Figure 8.**
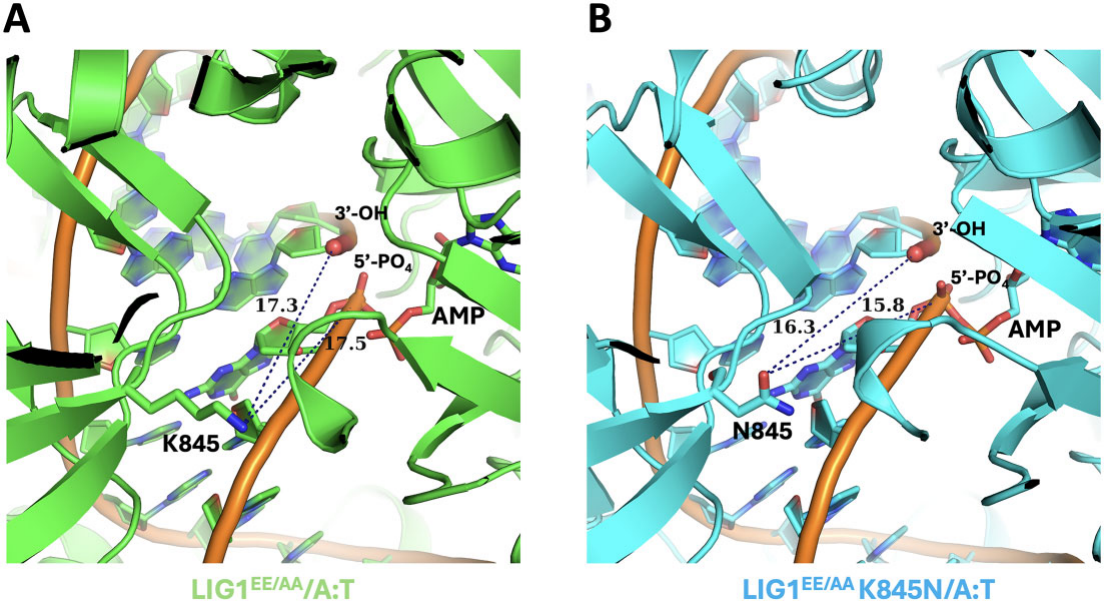
LIG1 structures demonstrate similarity in the distances relative to DNA ends. (**A** and **B**) Structures of LIG1^EE/AA^ and LIG1^EE/AA^K845N show the distances (>15 Å) between the ligase active site residues K845 versus N845 relative to the 3′-OH and 5′-PO_4_ ends of nick.

We then further analyzed the impact of the K845N mutation on the distances relative to the 3′- and 5′-ends of nick (Fig. 8). The distances between the functional groups of K845 and N845 side chains are calculated as 17.5 and 15.8 Å, respectively, relative to the 5′-end of nick. We observed a 1 Å difference in the distance between the K845/N845 functional group side chains with regards to the 3′-end of nick. These findings suggest that the LIG1^EE/AA^ and LIG1^EE/AA^ K845N structures share similar configuration with the nick site containing a canonical A:T, and therefore, the HD-associated amino acid substitution at the K845 residue likely does not impact nick processing at the ligase active site. As we crystalized the ligase (LIG1^EE/AA^) in the absence of Mg^2+^, the lack of the metal ion cofactor and the low-fidelity background could impact structural arrangements at the nick site when the ligation reaction proceeds. From the superimposition of the LIG1 structures (Supplementary Fig. S9), we conclude that the HD-associated variant K845N does not directly interact with the minor groove or template strand of the DNA as shown for previously solved LIG1 syndrome variants R641L and R771W [35].

### Nick DNA binding by LIG1 K845N variant at single-molecule level

**Figure 9.**
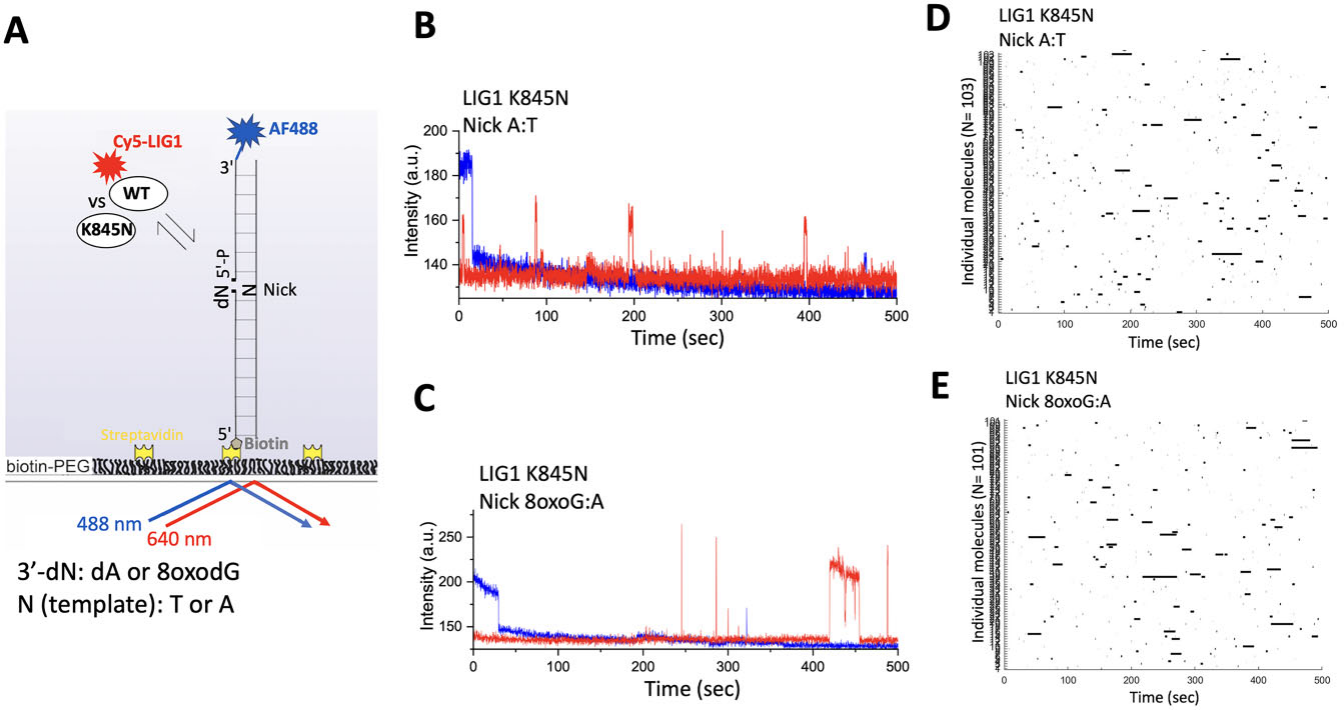
Single-molecule characterization of nick DNA binding by the LIG1 K845N variant. (**A**) Scheme shows that AF488-labeled dsDNA with a nick site immobilized on PEG-coated, biotinylated slide surface for imaging with a TIRF microscope to monitor real-time Cy5-labeled LIG1 (wild-type versus K845N) binding to nick. (**B–E**) Representative fluorescence intensity versus time traces and rastergrams of a randomly selected traces show repeated protein binding events by the LIG1 wild-type and K845N variant to nick DNA.

We employed a single-molecule fluorescence co-localization approach to monitor the impact of the LIG1 K845N mutation on nick DNA binding in real-time. For this purpose, we used the AF488-labeled dsDNA that contains a single nick site harboring a canonical A:T or damaged 8oxoG:A and Cy5-labeled LIG1 wild-type and K845N variant proteins to visualize LIG1^Cy5^ binding to nick by the co-localization of AF488 and Cy5 fluorescence signals within a diffraction-limited spot (Fig. 9A).

For both nick DNA substrates containing a canonical or damaged end, the analyses of individual fluorescence time trajectories showed repeated transient Cy5 co-localizations with AF488 for LIG1 wild-type and K845N variant (Fig. 9B and C). Similarly, the rastergrams generated from several individual time traces idealized by HMM also showed dynamic nick binding behavior for both LIG1 proteins (Fig. 9D and E, and Supplementary Fig. S10).

**Figure 10.**
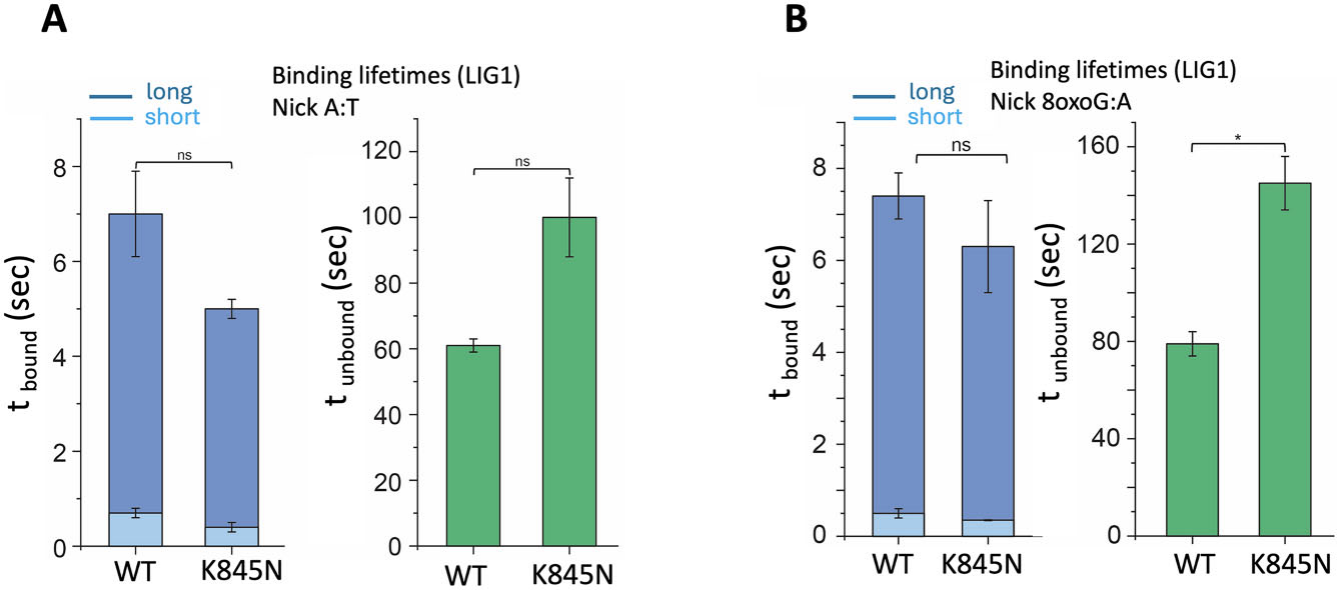
Comparison of nick DNA binding life-times between LIG1 wild-type and K845N variant for canonical and damaged nick substrates. (**A** and **B**) Bar graphs represent the comparison of *t*_bound_ and *t*_unbound_ times between LIG1 wild-type and K845N variant for nick DNA substrates containing canonical A:T and damaged 8oxoG:A ends. Data shown as mean ± SD from two independent experiments. *P-value < 0.05, n.s., not significant; Welch *t*-test.

We next estimated the average bound and unbound life-times from the dwell times distributions in the bound states (Fig. 10). For canonical nick DNA, we observed two populations with average binding life-times of 7.0 ± 0.9 s (0.4 ± 0.1%) and 0.7 ± 0.1 s (0.6 ± 0.1%) by LIG1 wild-type, which is consistent with our recent study [20]. For the LIG1 K845N variant, the average binding life-times were calculated as 5.0 ± 0.2 s (0.4 ± 0.01%) and 0.4 ± 0.1 s (0.6 ± 0.01) as shown in differences in *t*_bound_ and *t*_unbound_ times in the presence of canonical nick (Fig. 10A). The unbound time (*t*_unbound_) was slightly increased from 61 ± 2 s for LIG1 wild-type to 100 ± 12 s for LIG1 K845N variant. In the presence of nick substrate containing 8oxoG damage, our results showed no significant difference in *t*_bound_, but the *t*_unbound_ increased significantly from 79 ± 5 s for LIG1 wild-type to 145 ± 11 s for the K845N variant (Fig. 10B). We previously demonstrated that the long binding events for LIG1 and nick DNA interaction are due to the formation of a stable nick-bound LIG1–DNA complex [20]. This observation of less frequent LIG1 K845N binding to different nick substrates indicates that, compared to the LIG1 wild-type, K845N has less affinity to find a nick substrate. We did not observe a significant difference in *t*_bound_ and *t*_unbound_ times for both nick substrates (A:T and 8oxoG:A) and for each LIG1 protein (wild-type and K845N variant) as shown in the average binding life-times (Supplementary Figs S11 and S12).

In the control experiments, we performed single-molecule binding assays in the presence of dsDNA without a nick site and observed that both LIG1 proteins have similar bound and unbound time (Supplementary Figs S13 and 14). Interestingly, the comparison of binding parameters for dsDNA substrates with and without a nick site demonstrated, contrary to LIG1 wild-type, the bound and unbound times of the K845N variant are similar with average long *t*_bound_ of 5 ± 0.2 s and *t*_unbound_ of 100 ± 12 s for nick, whereas 4 ± 0.7 s and 113 ± 8 s for no-nick substrate. The observation of short-lived and less frequent binding events for LIG1 wild-type and no-nick substrate could be attributed to the nonspecific binding of the protein throughout the DNA [20]. Hence, the observation of similar binding characteristics for the LIG1 K845N binding to nick and no-nick substrates suggests that the mutant binds mostly nonspecifically through the DNA and forms a less stable DNA-bound complex.

Overall, the LIG1 K845N variant exhibits less frequent binding events and shorter binding life-time time, suggesting that the variant is less efficient at finding a target, and the stability of the nick DNA–LIG1 complex is relatively less. These results are consistent with the ligation assays where we demonstrated that the K845N mutation decreases ligation efficiency on the nick substrates containing canonical end (3′-dA:T), and increases discrimination against the nick containing a damaged end (3′-8oxodG:A) as shown by a ∼10-fold difference in the amount of ligation products between the LIG1 wild-type and K845N variant (Figs 1 and 2). This suggests that the LIG1 K845N variant binds to the nick with a reduced affinity and that further steps of the ligation reaction are adversely affected by K to N substitution in the presence of unusual ends at nick.

## Discussion

Unstable CAG expansion in the coding region of the *HTT* gene leads to mHTT production, with eventual aggregation resulting in HD that causes nerve cells in the brain to decay over time [26–29]. In a recent study [30], two alleles, CAA-loss and CAACAG-duplication mutants, were used to confirm the relationship between uninterrupted CAG repeat length and the disease onset, summarizing that the rate-driver of uninterrupted CAG repeats is independent of glutamine coding properties. This study also revealed that the K845N mutation residing in the OBD domain of LIG1 is predicted to be onset delaying and suppress CAG repeat expansion. LIG1, as the main replicative ligase, joins Okazaki fragments and seals the final nick product during the ultimate step of DNA excision repair pathways [6–11]. Yet, it remains unknown how the HD-associated hereditary LIG1 impairment due to the K845N mutation could impact ligase function at the final step. In the present study, we comprehensively characterized the impact of the LIG1 K845N variant on the ligase fidelity, DNA-binding affinity, and nick sealing efficiency at the biochemical, structural, and single-molecule levels.

Human immunodeficiency syndrome caused by the mutations in the *LIG1* gene (P529L, R641L, and R771W) leads to an inherited LIG1 deficiency, which was established from the individuals that exhibit growth retardation, developmental delays, sun sensitivity, and severe immunodeficiency [36–38]. The cells derived from the patients with LIG1 syndrome exhibit an aberrant joining of Okazaki fragments and hypersensitivity to DNA alkylating agents [39–41]. The amino acid residues that have been mutated in the LIG1 syndrome are located in the C-terminal catalytic domain of the protein, particularly in the AdD (E566), the DBD (P529) and the OBD (R771) domains (Supplementary Fig. S1A). Our previous study demonstrated that the ligation efficiency is compromised by LIG1 R641L and R771W variants for the nick substrates containing mismatches or 8oxoG, while LIG1 P529L variant shows wild-type level of end-joining ability to seal nicks with noncanonical ends [16].

Furthermore, X-ray structures of LIG1 variants R641L and R771W revealed a compromised cooperative network of interactions with the ligase active site and high-fidelity Mg^2+^-binding site leading to a decrease in DNA-binding affinity and ligation efficiency [35]. Particularly, R641L leads to a disengagement in the DNA-binding loop with connecting salt bridge to the active site (R641–D600 salt bridge anchor) as well as distortions in the DNA backbone, while LIG1 R771W causes remodeling of interdomain contacts at the juncture of the OBD and DBD domains, which is characterized by two salt bridges that support the OBD–DNA binding loop for stability in the minor groove. In their respective locations, both variants seem to disrupt the binding of protein–DNA–metal ion interactions that are usually involved in the mechanism by which LIG1 discriminates against nicks containing mismatch and oxidative damage through Mg^2+^ binding-mediated high-fidelity. From the overlay of previously solved structures of LIG1 syndrome variants R641L and R771W versus the HD-associated LIG1 variant K845N, the RMSD analysis suggest no significant changes in the overall structure (Supplementary Table S8). Furthermore, the superimposition of these structures also demonstrated that LIG1 R641L and R771W variants interact with the minor groove of the DNA, while the LIG1 K845N variant has no direct interaction with the DNA (Supplementary Fig. S9).

Our results from the ligation assays for a range of nick DNA substrates showed reduced nick sealing for all 12 possible noncanonical mismatches, and diminished mutagenic ligation of 8oxoG base paired with template A by the LIG1 K845N variant. Regarding DNA–RNA substrates containing a single ribonucleotide at the 3′-end of nick, our results demonstrated a lack of sugar discrimination, suggesting that the K845 side chain has no role for identification of “wrong” sugar at the nick. Furthermore, the overlay of the LIG1 structures in the presence and absence of the N845 mutation demonstrated similar conformations and only slight differences in the distances to both DNA ends. Further structural studies of the LIG1 K845N variant with nicks containing mismatched or damaged ends could provide more atomic insights into the mechanism by which the ligase active site carrying the HD-associated K845N mutation ensures ligase fidelity. In addition, our single-molecule measurements in real-time revealed that the LIG1 K845N variant binds to both canonical and damaged nick DNA less frequently when compared to the wild-type protein, although both proteins can form a stable nick-bound LIG1–DNA complex. Moreover, as indicated by similar binding parameters for nick and no-nick substrates, DNA binding by the LIG1 K845N variant is mostly nonspecific.

We previously reported that DNA binding by LIG1 is enriched near the nick sites and the protein exhibits diffusive behavior to form a long-lived ligase/nick complex after binding to a no-nick region [20]. Further single-molecule studies are required to visualize how the HD-associated K845N mutation could impact the mechanism of nick search by LIG1. Because the conversion of nicks into a phosphodiester bond requisite a rapid nick recognition to avoid deterioration of the exposed DNA ends to exonuclease degradation and increased frequency of recombination, this slower nick binding affinity we observed with the LIG1 K845N variant likely delays joining of SSBs and the maturation of Okazaki fragments. In the event of DNA replication, this situation may cause replication forks to stall, activating fork rescue pathways that could involve recombination-based mechanisms that do not favor repeat expansion [42]. This could potentially explain why the LIG1 K845N mutation causes delayed symptom onset and suppression of repeat expansion. It’s also important to note a functional redundancy between LIG1 and LIG3α in DNA repair and replication processes [43]. Furthermore, recent studies reported a back-up role of LIG3α in the case of perturbation to LIG1 leading to the formation of unligated Okazaki fragments during nuclear replication, which is trapped by poly(ADP-ribose) polymerase [44]. Therefore, LIG3α may compensate this inefficiency caused by the LIG1 K845N mutation during DNA replication or repair leading to less reliance on LIG1.

The molecular basis underlying trinucleotide repeat expansions such as age-dependent neuronal CAG repeats has been found to be associated with the formation of non-B-form or DNA noncanonical structures that can compromise proper function of DNA repair enzymes [45]. This may cause to the integration of hairpin intermediates or loops with different size and stability into the genome [46]. To further explore mechanisms of the repeat expansion caused by the LIG1 K845N mutation, more biochemical characterization assays are required to elucidate how this HD-associated mutation could impact the LIG1 function leading to the formation of strand slippage or flap equilibration during repair synthesis and how LIG1 K845N variant coordinate with other repair and replication proteins during processing of noncanonical DNA structures and hairpins. Overall, our study contributes to an understanding of how a defect in LIG1 ability to bind and join strand breaks, stemming from the K845N mutation, could lead to interruption in processing of a final nick product in normal versus disease states, underscoring how an aberrant LIG1 function could play a role in suppression of CAG repeat expansion and therefore delay HD-symptom onset.

## Supplementary Material

ugaf038_Supplemental_File

## Data Availability

Correspondence and requests for materials should be addressed to Melike Çağlayan (caglayanm@ufl.edu). Atomic coordinates and structure factors for the reported crystal structures of LIG1 K845N have been deposited in the RCSB Protein Data Bank under accession number of 9NYS. All relevant data are available from the authors upon reasonable request.

## Notes

Present address: Eppley Institute for Research in Cancer, Fred & Pamela Buffett Cancer Center, University of Nebraska Medical Center, Omaha, NE 68198, United States
