# Supplementary material for: Impaired nick recognition and ligation efficiency by LIG1 K845N variant linked to Huntington’s disease
Source: NAR Mol Med. 2025 Oct 28;2(4):ugaf038. doi: 10.1093/narmme/ugaf038 (PMC12673853; doi:10.1093/narmme/ugaf038)
Supplement: ugaf038_Supplemental_File [file ugaf038_supplemental_file.pdf]

Impaired nick recognition and ligation efficiency by LIG1 K845N variant linked to Huntington's Disease

Jacob Ratcliffe[1,*], Camden E. Lerner[1,*], Kanal Balu[1], Surajit Chatterjee[1], Kar Men Lee[1], Melike Çağlayan[1,#]

[1]Department of Biochemistry and Molecular Biology, University of Florida, Gainesville, FL 32610, USA

[*]The authors share co-first authorship

[#]Present address: Eppley Institute for Research in Cancer, Fred & Pamela Buffett Cancer Center, University of Nebraska Medical Center, Omaha, NE 68198

E-mail: mcaglayan@unmc.edu

Supplementary Figures 1-14

Supplementary Schemes 1-2

Supplementary Tables 1-8

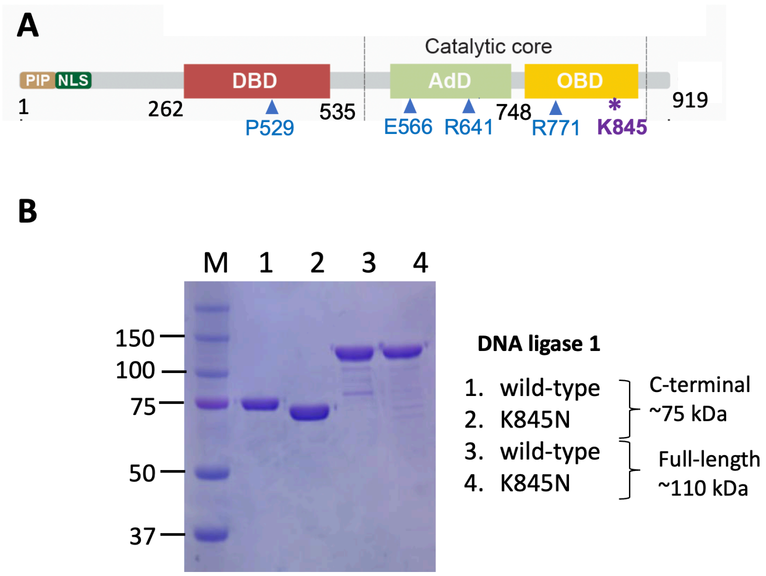

Supplementary Figure 1. LIG1 proteins used in the study. (A) The domain organization of DNA ligase 1 (LIG1) protein (1-919 amino acids) including N-terminal region (1-262 amino acids) and C-terminal catalytic core that contains DNA-binding domain (DBD) and the catalytic core consisting of Adenylation (AdD) and Oligonucleotide-binding (OBD) domains. The mutations at the amino acid residues that have been associated with LIG1 syndrome are located in the C-terminal catalytic domain of the protein, particularly in the DBD (P529), the AdD (E566, R641), and the OBD (R771) domains. LIG1 HD disease-asssociated mutation K845N resides in the OBD domain of the catalytic core. **(B)** SDS-PAGE gel shows the purity of LIG1 proteins used in this study. M represents a Precision Plus Protein Dual Color Standard (10-250 kDa).

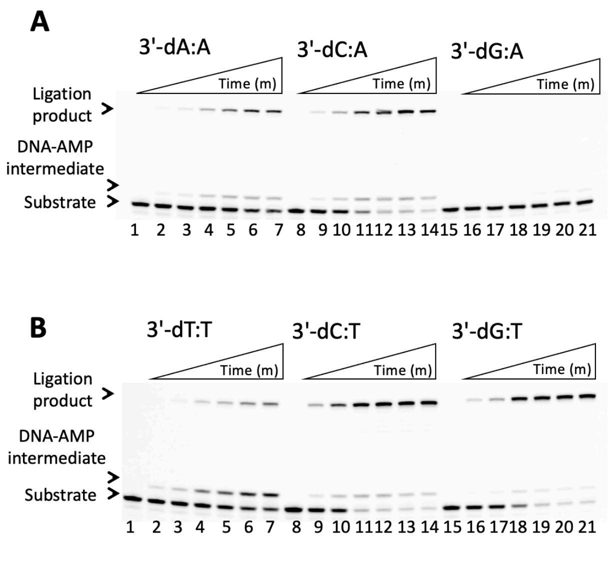

Supplementary Figure 2. Ligation efficiency of LIG1 wild-type for nick DNA substrates containing all possible 12 non-canonical mismatches. (A) Lanes 1, 8, and 15 are the negative enzyme controls of the nick DNA substrates with 3'-dA:A, 3'-dC:A, and 3'-dG:A mismatches, respectively. **(B)** Lanes 1, 8, and 15 are the negative enzyme controls of the nick DNA substrates with 3'-dT:T, 3'-dC:T, and 3'-dG:T mismatches, respectively. **(C)** Lanes 1, 8, and 15 are the negative enzyme controls of the nick DNA substrates with 3'-dG:G, 3'-dA:G, and 3'-dT:G mismatches, respectively. **(D)** Lanes 1, 8, and 15 are the negative enzyme controls of the nick DNA substrates with 3'-dC:C, 3'-dA:C, and 3'-dT:C mismatches, respectively. In all panels, lanes 2-7, 9-14, and 16-21 are the ligation reaction products by LIG1 wild-type, and correspond to time points of 0.5, 1, 3, 5, 8, and 10 min. Graphs show time-dependent changes in the amount of ligation products are presented in Figure 3.

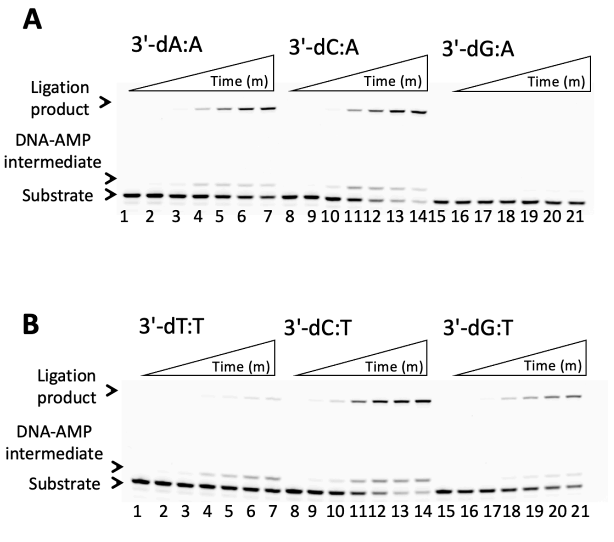

Supplementary Figure 3. Ligation efficiency of LIG1 K845N variant for nick DNA substrates containing all possible 12 non-canonical mismatches. (A) Lanes 1, 8, and 15 are the negative enzyme controls of the nick DNA substrates with 3'-dA:A, 3'-dC:A, and 3'-dG:A mismatches, respectively. **(B)** Lanes 1, 8, and 15 are the negative enzyme controls of the nick DNA substrates with 3'-dT:T, 3'-dC:T, and 3'-dG:T mismatches, respectively. **(C)** Lanes 1, 8, and 15 are the negative enzyme controls of the nick DNA substrates with 3'-dG:G, 3'-dA:G, and 3'-dT:G mismatches, respectively. **(D)** Lanes 1, 8, and 15 are the negative enzyme controls of the nick DNA substrates with 3'-dC:C, 3'-dA:C, and 3'-dT:C mismatches, respectively. In all panels, lanes 2-7, 9-14, and 16-21 are the ligation reaction products by LIG1 K845N mutant, and correspond to time points of 0.5, 1, 3, 5, 8, and 10 min. Graphs show time-dependent changes in the amount of ligation products are presented in Figure 3.

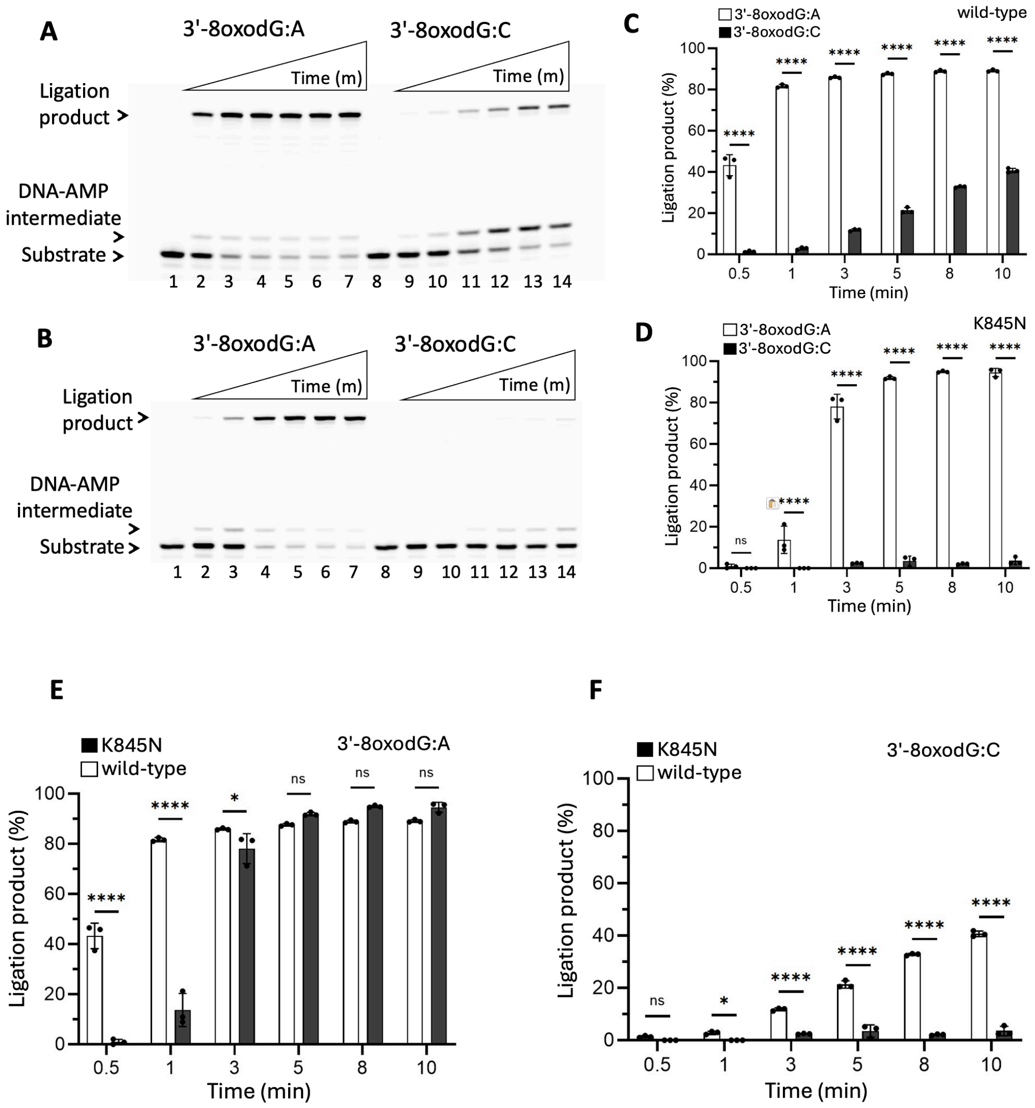

Supplementary Figure 4. Ligation efficiency of LIG1 wild-type and K845N variant for nick DNA substrates containing damaged ends. (A-B) Lanes 1 and 8 are the negative enzyme controls of the nick DNA substrates with 3'-8oxodG:A and 3'-8oxodG:C, respectively. In both panels, lanes 2-7 and 9-14 are the ligation reaction products by LIG1 wild-type (A) and K845N variant (B), and correspond to time points of 0.5, 1, 3, 5, 8, and 10 min. **(C-F)** Graphs show time-dependent changes in the amount of ligation products. Data points represent three independent replicates. Bar height is the mean, and error bars represent the SD. n.s., not significant; *P < 0.05, ****P<0.0001 by ordinary two-way ANOVA with multiple comparisons.

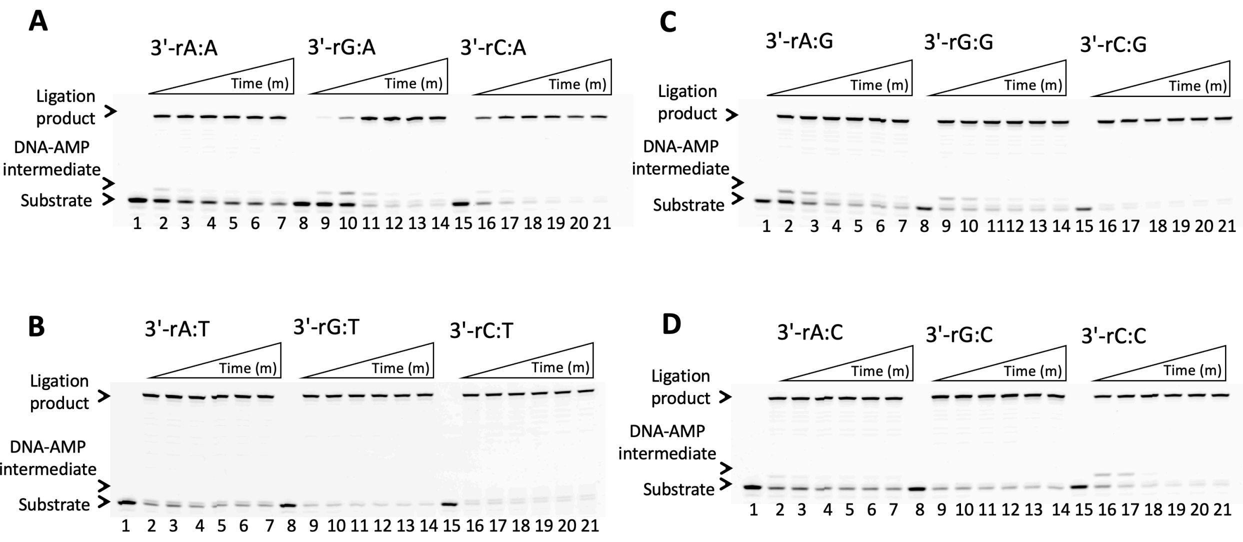

Supplementary Figure 5. Ligation efficiency of LIG1 wild-type for nick DNA substrates containing 3'-ribonucleotides. (A) Lanes 1, 8, and 15 are the negative enzyme controls of the nick DNA substrates with 3'-rA:A, 3'-rG:A, and 3'-rC:A, respectively. **(B)** Lanes 1, 8, and 15 are the negative enzyme controls of the nick DNA substrates with 3'-rA:T, 3'-rG:T, and 3'-rC:T, respectively. In both panels, lanes 2-7, 9-14, and 16-21 are the ligation reaction products by LIG1 wild-type, and correspond to time points of 0.5, 1, 3, 5, 8, and 10 min. **(C)** Lanes 1, 8, and 15 are the negative enzyme controls of the nick DNA substrates with 3'-rA:G, 3'-rG:G, and 3'-rC:G, respectively. **(D)** Lanes 1, 8, and 15 are the negative enzyme controls of the nick DNA substrates with 3'-rA:C, 3'-rG:C, and 3'-rC:C, respectively. In all panels, lanes 2-7, 9-14, and 16-21 are the ligation reaction products by LIG1 wild-type, and correspond to time points of 0.5, 1, 3, 5, 8, and 10 min. Graphs show time-dependent changes in the amount of ligation products are presented in Figure 4.

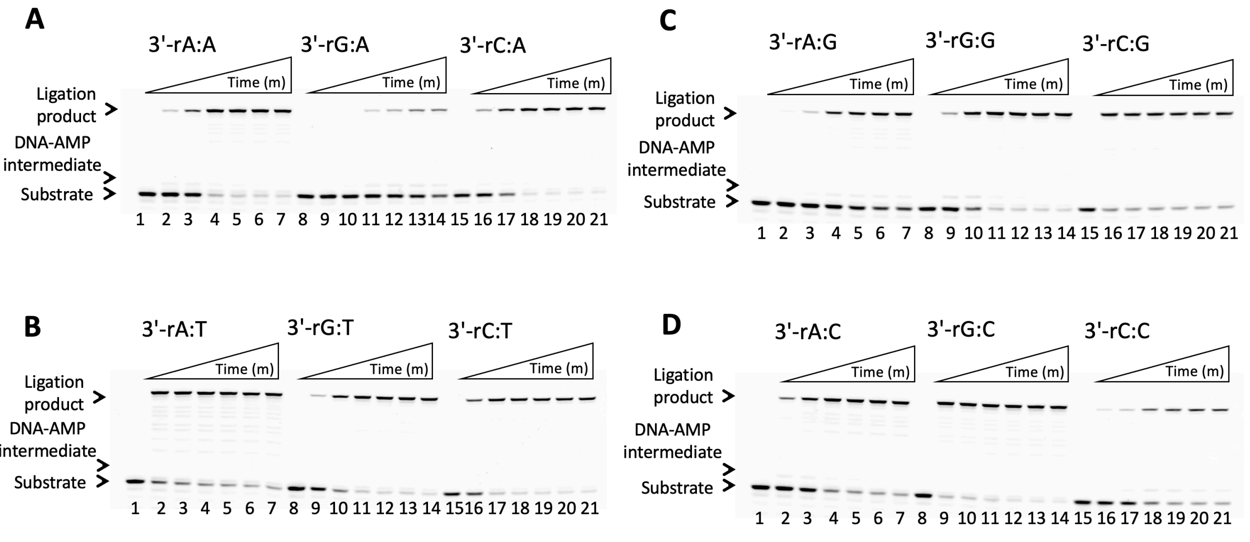

Supplementary Figure 6. Ligation efficiency of LIG1 K845N variant for nick DNA substrates containing 3'-ribonucleotides. (A) Lanes 1, 8, and 15 are the negative enzyme controls of the nick DNA substrates with 3'-rA:A, 3'-rG:A, and 3'-rC:A, respectively. **(B)** Lanes 1, 8, and 15 are the negative enzyme controls of the nick DNA substrates with 3'-rA:T, 3'-rG:T, and 3'-rC:T, respectively. In both panels, lanes 2-7, 9-14, and 16-21 are the ligation reaction products by LIG1 wild-type, and correspond to time points of 0.5, 1, 3, 5, 8, and 10 min. **(C)** Lanes 1, 8, and 15 are the negative enzyme controls of the nick DNA substrates with 3'-rA:G, 3'-rG:G, and 3'-rC:G, respectively. **(D)** Lanes 1, 8, and 15 are the negative enzyme controls of the nick DNA substrates with 3'-rA:C, 3'-rG:C, and 3'-rC:C, respectively. In all panels, lanes 2-7, 9-14, and 16-21 are the ligation reaction products by LIG1 K845N variant, and correspond to time points of 0.5, 1, 3, 5, 8, and 10 min. Graphs show time-dependent changes in the amount of ligation products are presented in Figure 4.

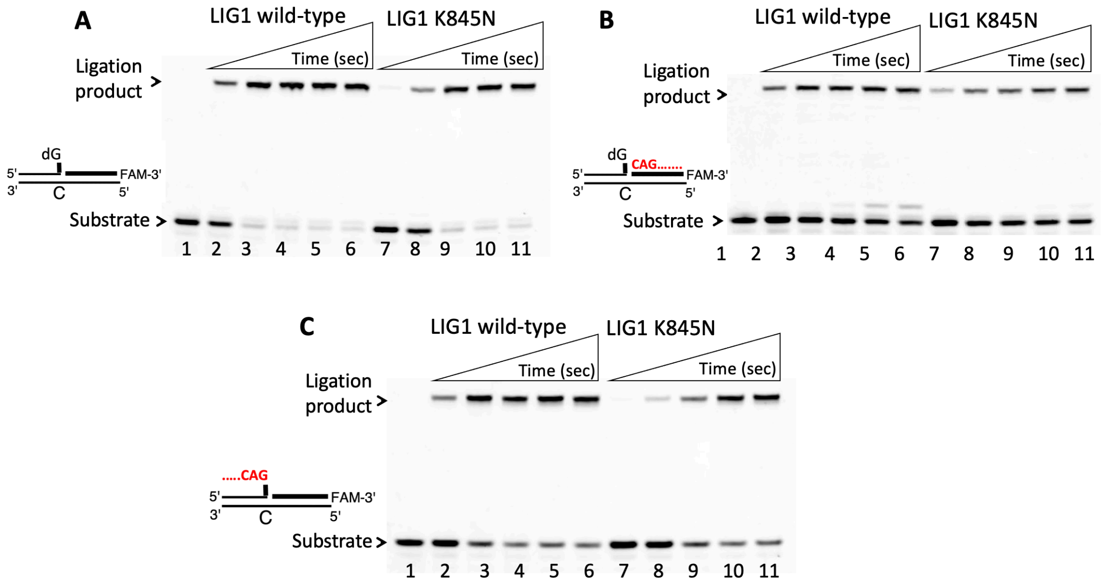

Supplementary Figure 7. Ligation efficiency of LIG1 K845N variant for nick DNA substrates containing CAG repeats. (A-C) Line 1 is the negative enzyme controls of the nick DNA substrates containing 3'-dG:C (without CAG repeat sequence), 3'-dG:C (with CAG repeat sequence at upstream position), and 3'-dG:C (with CAG repeat sequence at downstrean position). Lanes 2-6 and 8-12 are the ligation reaction products by LIG1 wild-type and K845N mutant, respectively, and correspond to time points of 10, 30, 60, 90, 120 sec. Graphs show time-dependent changes in the amount of ligation products are presented in Figure 5.

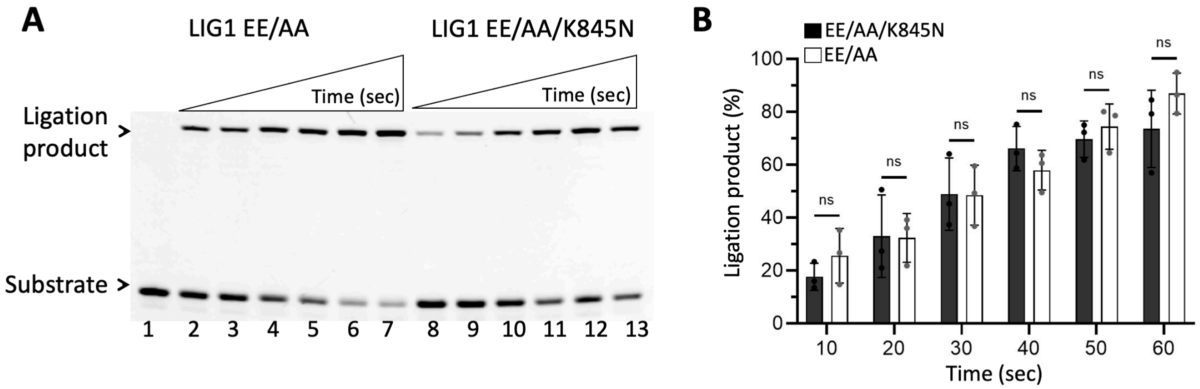

Supplementary Figure 8. Ligation efficiency of LIG1 K845N variant in the low-fidelity background. (A) Line 1 is the negative enzyme control of the nick DNA substrate with 3'-dA:T. Lanes 2-7 and 8-13 are the ligation reaction products by LIG1 EE/AA double mutant and LIG1 EE/AA/K845N triple-mutant, respectively, and correspond to time points of 10, 20, 30, 40, 50, and 60 sec. **(B)** Graph shows time-dependent changes in the amount of ligation products. Data points represent three independent replicates. Bar height is the mean, and error bars represent the SD. n.s., not significant by ordinary two-way ANOVA with multiple comparisons.

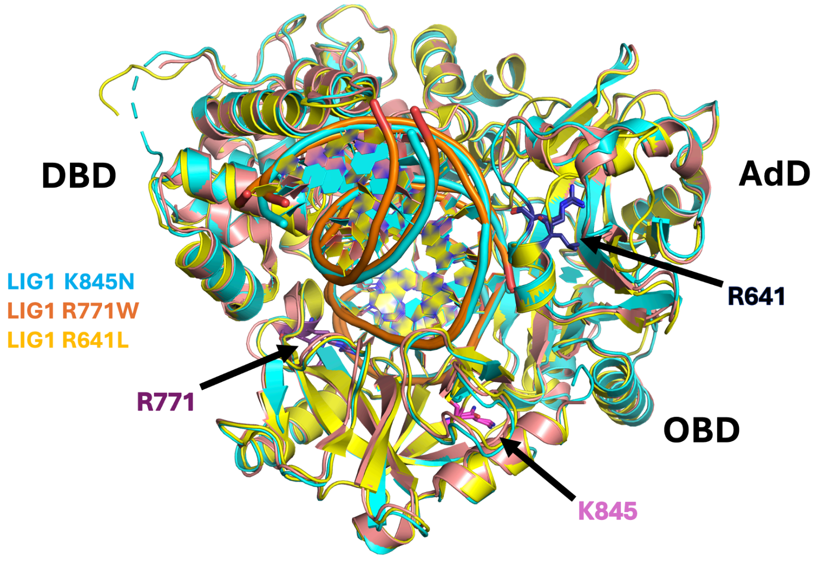

Supplementary Figure 9. Superimposition of LIG1 disease mutant structures. The overlay of LIG1 structures for LIG1 syndrome mutants R641L (blue) and R771W (purple) with HD-associated variant K845N (cyan) in the presence of nick containing canonical end reveals that the LIG1 syndrome mutants directly interacts with the minor groove/template strand of the DNA, while the K845N does not interact with the minor or major groove of the DNA. LIG1 syndrome mutations are located in the AdD (R641L) and the OBD (R771W) domains, while LIG1 HD-asssociated K845N mutant resides in OBD domain of LIG1. The structures of LIG1 syndrome mutants were previously solved by other group (35) for LIG1^{R641L} (7L34) and LIG1^{R771W} (7L35).

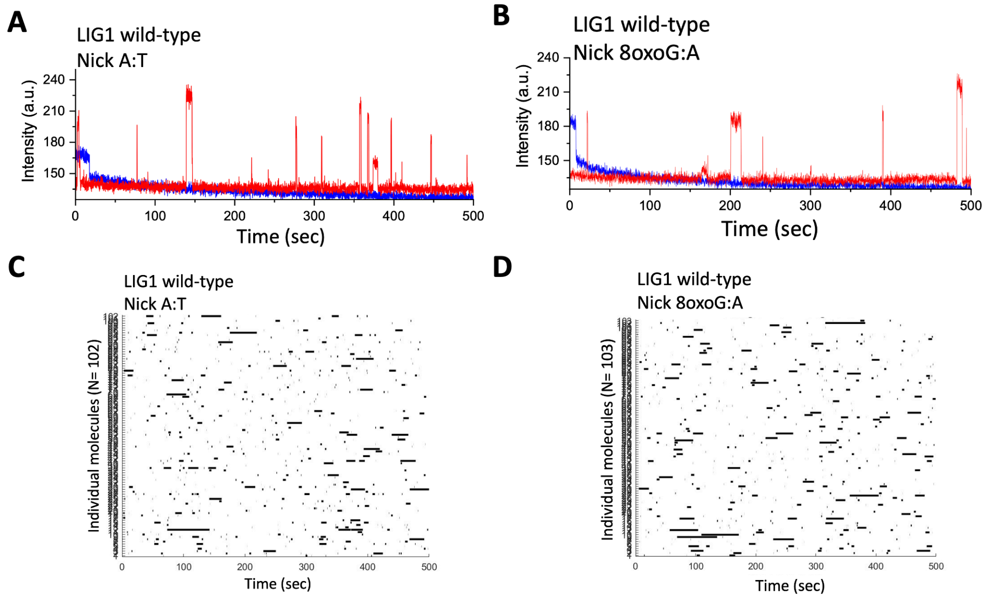

Supplementary Figure 10. Nick DNA binding by LIG1 wild-type. (A-B) Fluorescence intensity *versus* time traces show repeated protein binding events of LIG1 wild-type to nick containing canonical (A) and damaged (B) ends. **(C-D)** Rastergrams of randomly selected traces are shown for LIG1 wild-type protein displaying the distinct nick DNA binding behavior in the presence of nick substrates containing canonical A:T (C) and damaged 8oxoG:A (D) ends.

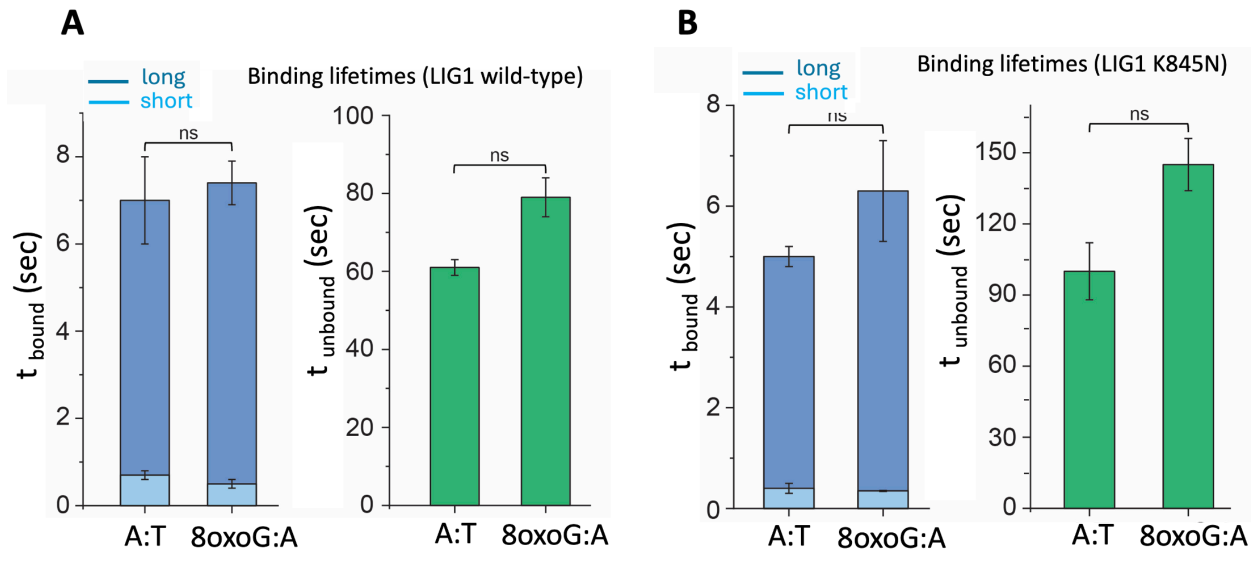

Supplementary Figure 11. Comparison of DNA binding by LIG1 K845N variant to nick containing canonical *versus* damaged ends. (A-B) Bar graphs represent the differences in life-times of protein-bound and -unbound states and show the comparison of t_{bound} and $t_{un\text{bound}}$ times between LIG1 wild-type and K845N variant for dsDNA substrates containing canonical A:T *versus* damaged 8oxoG:A ends.

A

Nick A:T

LIG1	t_{bound}, s (amplitude)	$t_{unbound}$, s (100%)	*N*
wild-type	7 ± 0.9 (0.4 ± 0.1),<br>0.7 ± 0.1 (0.6 ± 0.1)	61 ± 2	206
K845N	5 ± 0.2 (0.4 ± 0.01),<br>0.4 ± 0.1 (0.6 ± 0.01)	100 ± 12	206

B

Nick 8oxoG:A

LIG1	t_{bound}, s (amplitude)	$t_{unbound}$, s (100%)	*N*
wild-type	0.5 ± 0.1 (0.53 ± 0.01),<br>7.4 ± 0.5 (0.47 ± 0.01)	79 ± 5	205
K845N	0.35 ± 0.01 (0.6 ± 0.04),<br>6.3 ± 1 (0.4 ± 0.04)	145 ± 11	204

Supplementary Figure 12. Comparison of nick DNA binding by LIG1 wild-type and K845N variant to nicks containing canonical *versus* damaged ends. (A-B) Tables show the differences in the protein-bound (t_{bound}) and unbound ($t_{un\text{bound}}$) states that represent the binding life-times of LIG1 wild-type and K845N variant to nick DNA substrates containing canonical (A) and damaged (B) ends.

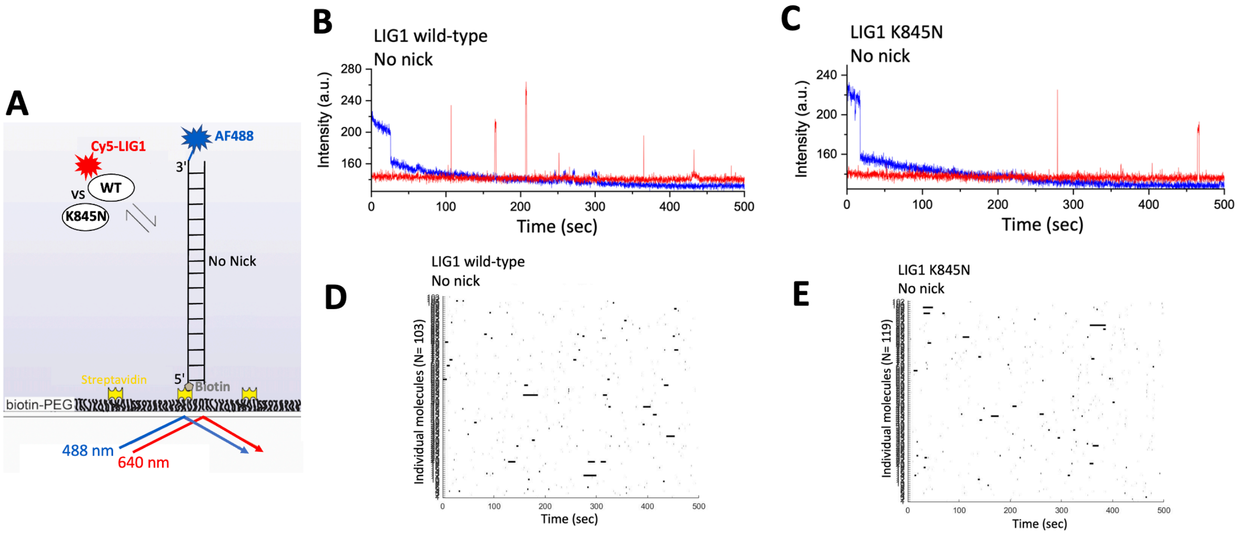

F

No nick

LIG1	t_{bound}, s (amplitude)	$t_{unbound}$, s (100%)	*N*
wild-type	0.3 ± 0.04 (0.8), 3.4 ± 0.1 (0.2)	112 ± 4	105
K845N	0.2 ± 0.01 (0.8 ± 0.01), 4 ± 0.7 (0.2 ± 0.01)	113 ± 8	104

Supplementary Figure 13. DNA binding modes of LIG1 wild-type and K845N variant in the absence of nick. (A) Scheme shows that AF488-labeled dsDNA without a nick site immobilized on PEG-coated, biotinylated slide surface for imaging with a TIRF microscope to monitor real-time Cy5-labeled LIG1/DNA binding. **(B-C)** Fluorescence intensity *versus* time traces show repeated protein binding events of LIG1 wild-type and K845N variant to dsDNA without a nick site. **(D-E)** Rastergrams of randomly selected traces are shown for LIG1 wild-type and K845N variant proteins displaying the distinct DNA binding behavior in the absence of nick. **(F)** Table shows the differences in the protein-bound (t_{bound}) and unbound ($t_{unbound}$) states that represent the binding lifetimes of LIG1 wild-type and K845N variant to dsDNA without a nick site.

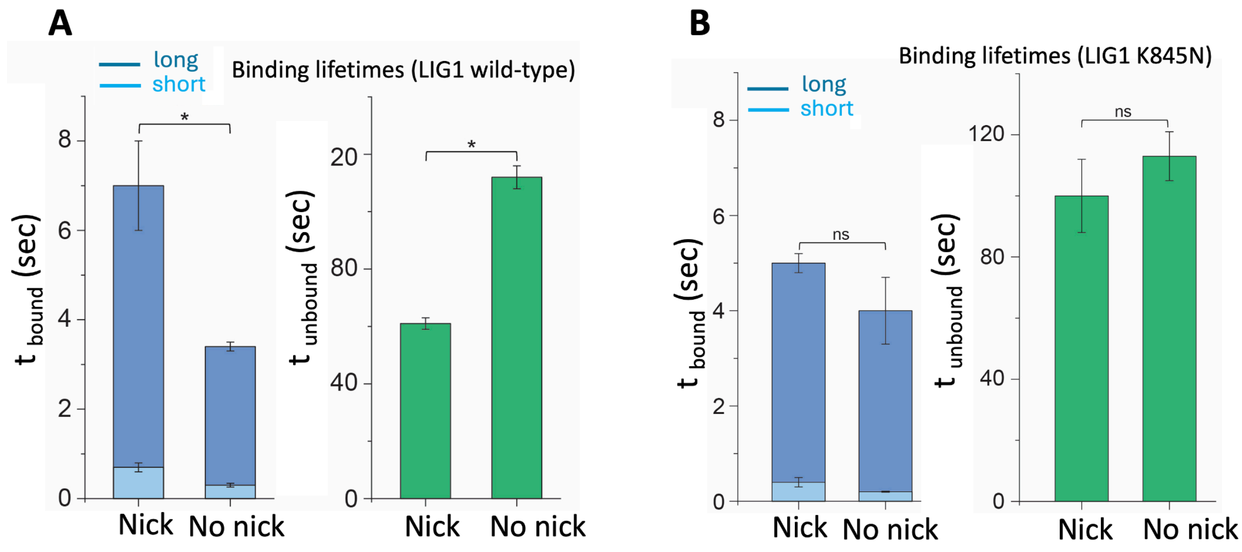

Supplementary Figure 14. Comparison of DNA binding by LIG1 wild-type and K845N variant in the absence and presence of nick site. (A-B) Bar graphs represent the differences in life-times of protein-bound and -unbound states and show the comparison of t_{bound} and $t_{un\text{bound}}$ times between LIG1 wild-type and K845N variant for dsDNA substrates containing a nick site *versus* no nick site.

Nick DNA Substrates	Sequence
3'-dA:A	5'-CATGGGCGGCATGAACCAGAGGCCCATCCTCACC-3'-FAM<br>3'-GTACCCGCCGTACTTGGACTCCGGGTAGGAGTGG-5'
3'-dG:A	5'-CATGGGCGGCATGAACCGGAGGCCCATCCTCACC-3'-FAM<br>3'-GTACCCGCCGTACTTGGACTCCGGGTAGGAGTGG-5'
3'-dC:A	5'-CATGGGCGGCATGAACCCGAGGCCCATCCTCACC-3'-FAM<br>3'-GTACCCGCCGTACTTGGACTCCGGGTAGGAGTGG-5'
3'-dT:A	5'-CATGGGCGGCATGAACCTGAGGCCCATCCTCACC-3'-FAM<br>3'-GTACCCGCCGTACTTGGACTCCGGGTAGGAGTGG-5'
3'-dT:T	5'-CATGGGCGGCATGAACCTGAGGCCCATCCTCACC-3'-FAM<br>3'-GTACCCGCCGTACTTGGTCTCCGGGTAGGAGTGG-5'
3'-dG:T	5'-CATGGGCGGCATGAACCGGAGGCCCATCCTCACC-3'-FAM<br>3'-GTACCCGCCGTACTTGGTCTCCGGGTAGGAGTGG-5'
3'-dC:T	5'-CATGGGCGGCATGAACCCGAGGCCCATCCTCACC-3'-FAM<br>3'-GTACCCGCCGTACTTGGTCTCCGGGTAGGAGTGG-5'
3'-dA:T	5'-CATGGGCGGCATGAACCAGAGGCCCATCCTCACC-3'-FAM<br>3'-GTACCCGCCGTACTTGGTCTCCGGGTAGGAGTGG-5'
3'-dA:G	5'-CATGGGCGGCATGAACCAGAGGCCCATCCTCACC-3'-FAM<br>3'-GTACCCGCCGTACTTGGGCTCCGGGTAGGAGTGG-5'
3'-dT:G	5'-CATGGGCGGCATGAACCTGAGGCCCATCCTCACC-3'-FAM<br>3'-GTACCCGCCGTACTTGGGCTCCGGGTAGGAGTGG-5'
3'-dG:G	5'-CATGGGCGGCATGAACCGGAGGCCCATCCTCACC-3'-FAM<br>3'-GTACCCGCCGTACTTGGGCTCCGGGTAGGAGTGG-5'
3'-dC:G	5'-CATGGGCGGCATGAACCCGAGGCCCATCCTCACC-3'-FAM<br>3'-GTACCCGCCGTACTTGGGCTCCGGGTAGGAGTGG-5'
3'-dA:C	5'-CATGGGCGGCATGAACCAGAGGCCCATCCTCACC-3'-FAM<br>3'-GTACCCGCCGTACTTGGCCTCCGGGTAGGAGTGG-5'
3'-dT:C	5'-CATGGGCGGCATGAACCTGAGGCCCATCCTCACC-3'-FAM<br>3'-GTACCCGCCGTACTTGGCCTCCGGGTAGGAGTGG-5'
3'-dC:C	5'-CATGGGCGGCATGAACCCGAGGCCCATCCTCACC-3'-FAM<br>3'-GTACCCGCCGTACTTGGCCTCCGGGTAGGAGTGG-5'
3'-dG:C	5'-CATGGGCGGCATGAACCGGAGGCCCATCCTCACC-3'-FAM<br>3'-GTACCCGCCGTACTTGGCCTCCGGGTAGGAGTGG-5'

Supplementary Table 1. Nick DNA substrates containing 3'-mismatches. Nick DNA substrates with 3'-preinserted dA, dT, dG, dC opposite template base A, T, G, or C were used in the ligation assays to investigate the mismatch specificity of LIG1 wild-type and K845N variant. FAM denotes a fluorescent tag and is located at 3'-end of DNA substrates. The base at the template position is underlined.

Nick DNA Substrates	Sequence
3'-8oxodG:A	5'-CATGGGCGGCATGAACC**X**GAGGCCCATCCTCACC-3'-FAM<br>3'-GTACCCGCCGTACTTGGACTCCGGGTAGGAGTGG-5'
3'-8oxodG:C	5'-CATGGGCGGCATGAACC**X**GAGGCCCATCCTCACC-3'-FAM<br>3'-GTACCCGCCGTACTTGGCCTCCGGGTAGGAGTGG-5'

Supplementary Table 2. Nick DNA substrates containing damaged ends. Nick DNA substrates with 3'-8-oxodG opposite template base A or C were used in the ligation assays to investigate the ligation efficiency of LIG1 wild-type and K845N variant. FAM denotes a fluorescent tag and is located at 3'-end of DNA substrates. The base at the template position is underlined. The damaged base is shown in bold.

Nick DNA Substrates	Sequence
3'-rA:A	5'-CATGGGCGGCATGAACC**A**GAGGCCCATCCTCACC-3'-FAM<br>3'-GTACCCGCCGTACTTGGACTCCGGGTAGGAGTGG-5'
3'-rG:A	5'-CATGGGCGGCATGAACC**G**GAGGCCCATCCTCACC-3'-FAM<br>3'-GTACCCGCCGTACTTGGACTCCGGGTAGGAGTGG-5'
3'-rC:A	5'-CATGGGCGGCATGAACC**C**GAGGCCCATCCTCACC-3'-FAM<br>3'-GTACCCGCCGTACTTGGACTCCGGGTAGGAGTGG-5'
3'-rG:T	5'-CATGGGCGGCATGAACC**G**GAGGCCCATCCTCACC-3'-FAM<br>3'-GTACCCGCCGTACTTGGTCTCCGGGTAGGAGTGG-5'
3'-rC:T	5'-CATGGGCGGCATGAACC**C**GAGGCCCATCCTCACC-3'-FAM<br>3'-GTACCCGCCGTACTTGGTCTCCGGGTAGGAGTGG-5'
3'-rA:T	5'-CATGGGCGGCATGAACC**A**GAGGCCCATCCTCACC-3'-FAM<br>3'-GTACCCGCCGTACTTGGTCTCCGGGTAGGAGTGG-5'
3'-rA:G	5'-CATGGGCGGCATGAACC**A**GAGGCCCATCCTCACC-3'-FAM<br>3'-GTACCCGCCGTACTTGGGCTCCGGGTAGGAGTGG-5'
3'-rG:G	5'-CATGGGCGGCATGAACC**G**GAGGCCCATCCTCACC-3'-FAM<br>3'-GTACCCGCCGTACTTGGGCTCCGGGTAGGAGTGG-5'
3'-rC:G	5'-CATGGGCGGCATGAACC**C**GAGGCCCATCCTCACC-3'-FAM<br>3'-GTACCCGCCGTACTTGGGCTCCGGGTAGGAGTGG-5'
3'-rA:C	5'-CATGGGCGGCATGAACC**A**GAGGCCCATCCTCACC-3'-FAM<br>3'-GTACCCGCCGTACTTGGCCTCCGGGTAGGAGTGG-5'
3'-rC:C	5'-CATGGGCGGCATGAACC**C**GAGGCCCATCCTCACC-3'-FAM<br>3'-GTACCCGCCGTACTTGGCCTCCGGGTAGGAGTGG-5'
3'-rG:C	5'-CATGGGCGGCATGAACC**G**GAGGCCCATCCTCACC-3'-FAM<br>3'-GTACCCGCCGTACTTGGCCTCCGGGTAGGAGTGG-5'

Supplementary Table 3. Nick DNA substrates containing 3'-ribonucleotides. Nick DNA substrates with 3'-preinserted rA, rG, rC opposite template base A, T, G, or C were used in the ligation assays to investigate the sugar discrimination of LIG1 wild-type and K845N variant against nick DNA substrates congaining 3'-ribonucleotide. FAM denotes a fluorescent tag and is located at 3'-end of DNA substrates. The base at the template position is underlined. The ribonucleotide at 3'-end of nick is shown in bold.

Nick DNA Substrates	Sequence
3'-dG:C<br>(without CAG repeats)	5'-CATGGGCGGCATGAACCGGAGGCCCATCCTCACC-3'-FAM<br>3'-GTACCCGCCGTACTTGGCCTCCGGGTAGGAGTGG-5'
3'-dG:C<br>(with CAG repeats at upstream position)	5'-**CAG CAG CAG CAG CAG CAG** GAG GCC CAT CCT CACC-3'-FAM<br>3'-GTC GTC GTC GTC GTC GTC CTC CGG GTA GGA GTGG-5'
3'-dG:C<br>(with CAG repeats at downstream position)	5'- CAT GGG CGG CAT GAA CCG **CAG CAG CAG CAG CAG**-3'-FAM<br>3'- GTA CCC GCC GTA CTT GGC GTC GTC GTC GTC GTC-5'

Supplementary Table 4. Nick DNA substrates containing CAG repeats. Nick DNA substrates with 3'-dG:C with and without CAG repeat sequences relative to the 3'-end of nick position were used to compare the ligation efficiency of LIG1 wild-type and K845N mutant. FAM denotes a fluorescent tag and is located at 3'-end of DNA substrates. The bases at 3'-end of nick and the template position are underlined. The seqeuence containing CAG repeats are shown in bold.

Oligonucleotide	Sequence (5'-3')
Template T	GTCCGACTACGCATCAGC
Upstream A	GCTGATGCGT**A**
Downstream (5'-P)	P-GTCGGAC

Supplementary Table 5. Oligonucleotides used in LIG1 crystallization. Upstream oligonucleotide (3'-A), downstream oligonucleotide with phosphate (P) at the 5'-end, and template oligonucleotide containing T on a template position were used to prepare the nick DNA substrate with 3'-A:T for LIG1 crystallizations. The base at template base position is underlined and the base position at the 3'-end of nick is shown in bold.

Protein	Crystal Conditions	Cryo-protectant
LIG1$^{\text{EE/AA}}$	100 mM MES (pH 6.5), 200 mM Lithium acetate, 12% (w/v) PEG3350	1:1:1 of DMSO:Glycerol:PEG400
LIG1$^{\text{EE/AA/K845N}}$	100 mM MES (pH 6.0), 150 mM Lithium acetate, 12% (w/v) PEG3350	1:1:1 of DMSO:Glycerol:PEG400

Supplementary Table 6. Crystallization conditions of LIG1. LIG1-nick DNA complex crystals for LIG1$^{\text{EE/AA}}$ and LIG1$^{\text{EE/AA/K845N}}$ proteins were submerged in the cryoprotectant solution containing reservoir solution mixed with glycerol to a final concentration of 20% glycerol.

Oligonucleotide	Sequence
Up^{A}	5'-Bio-CATGGGCGGCATGAACC**A**-3'
Up$^{\text{8oxoG}}$	5'-Bio-CATGGGCGGCATGAACC**X**-3'
Template T	5'-GGTGAGGATGGGCCTCTGGTTCATGCCGCCCATG-3'
Template A	5'-GGTGAGGATGGGCCTCAGGTTCATGCCGCCCATG-3'
Down	5'-(P)GAGGCCCATCCTCACC-AF488-3'
No nick	5'-Bio-CATGGGCGGCATGAACCAGAGGCCCATCCTCACC-AF488-3'

Supplementary Table 7. Nick DNA substrates used in single-molecule DNA binding of LIG1 by TIRF. Up^{A}, Template T, and downstream oligonucleotides were used to prepare nick DNA substrate with canonical A:T end. Up$^{\text{8oxoG}}$, Template A, and downstream oligonucleotides were used to prepare nick DNA substrate with damaged 8oxoG:A end. No nick and Template T oligonucleotides were used to prepare the DNA substrate without a nick site. Bio denotes a Biotin label located at 5'-end, AF488 is a green-fluorescent dye located at 3'-end, and P stands for a phosphate at 5'-end. The base at 3'-end is shown as bold and the template base is underlined.

RMSD (Å)	LIG1$^{EE/AA}$	LIG1 R771W	LIG1 R641L
LIG1$^{EE/AA}$ K845N	0.558 (600)	0.567 (582)	0.463 (570)
LIG1$^{EE/AA}$		0.724 (0.589)	0.654 (576)
LIG1 R771W			0.544 (548)

Supplementary Table 8. RMSD values of LIG1 disease mutants. Table shows RMSD values for the structure of LIG1$^{EE/AA}$ K845N solved in the present study and the structures of LIG1 syndrome mutants that were previously solved by other group (50) for LIG1^{R641L} (7L34) and LIG1^{R771W} (7L35).Values in the parenthesis represents the number of atoms aligned against.

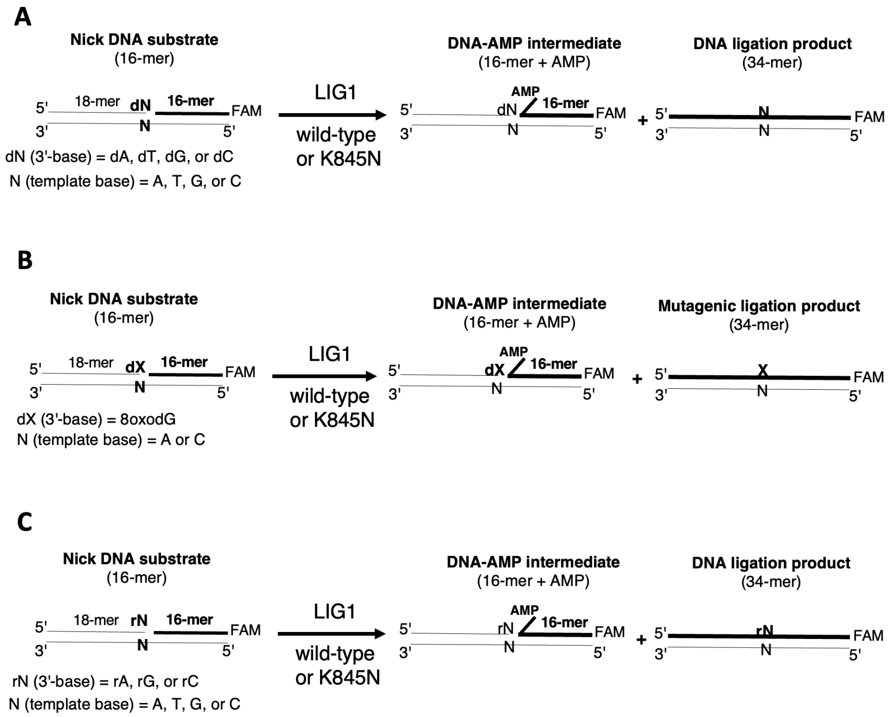

Supplementary Scheme 1. Illustrations of DNA ligation assays for nick substrates containing mismatch, damage, and rironucleotide. (A-C) Ligation assays were used to evaluate the substrate specificity of LIG1 wild-type and K845N variant for the nick DNA substrates including 3'-mismatches (A), 3'-8oxodG (B), and 3'-ribonucleotide (C). Reaction products include ligation and DNA-AMP intermediate with 5'-adenylate (AMP).

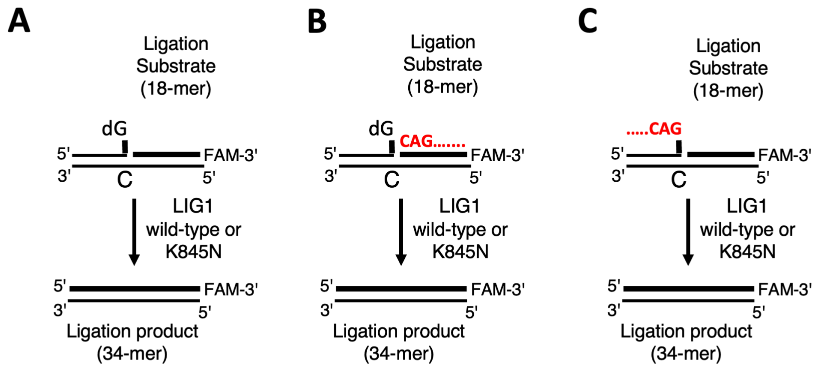

Supplementary Scheme 2. Illustrations of DNA ligation assays for nick substrates containing CAG repeats. (A-C) Ligation assays were used to evaluate the ligation efficiency of LIG1 wild-type and K845N variant for the nick DNA substrates containing canonical 3'-dG:C without CAG repeat sequence (A), with CAG repeat sequence at downstream (B) and upstream (C) position relative to nick site.
